# A dominant-negative mutant inhibits multiple prion variants through a common mechanism

**DOI:** 10.1371/journal.pgen.1007085

**Published:** 2017-10-30

**Authors:** Fen Pei, Susanne DiSalvo, Suzanne S. Sindi, Tricia R. Serio

**Affiliations:** 1 The University of Arizona, Department of Molecular and Cellular Biology, Tucson, Arizona, United States of America; 2 Brown University, Department of Molecular and Cell Biology, Providence, Rhode Island, United States of America; 3 University of California, Merced, Applied Mathematics, School of Natural Sciences, Merced, California, United States of America; University of Kent, UNITED KINGDOM

## Abstract

Prions adopt alternative, self-replicating protein conformations and thereby determine novel phenotypes that are often irreversible. Nevertheless, dominant-negative prion mutants can revert phenotypes associated with some conformations. These observations suggest that, while intervention is possible, distinct inhibitors must be developed to overcome the conformational plasticity of prions. To understand the basis of this specificity, we determined the impact of the G58D mutant of the Sup35 prion on three of its conformational variants, which form amyloids in *S*. *cerevisiae*. G58D had been previously proposed to have unique effects on these variants, but our studies suggest a common mechanism. All variants, including those reported to be resistant, are inhibited by G58D but at distinct doses. G58D lowers the kinetic stability of the associated amyloid, enhancing its fragmentation by molecular chaperones, promoting Sup35 resolubilization, and leading to amyloid clearance particularly in daughter cells. Reducing the availability or activity of the chaperone Hsp104, even transiently, reverses curing. Thus, the specificity of inhibition is determined by the sensitivity of variants to the mutant dosage rather than mode of action, challenging the view that a unique inhibitor must be developed to combat each variant.

## Author summary

Prion proteins adopt alternative conformations and assemble into amyloid fibers, which have been associated with human disease. These fibers are highly stable and self-replicate, leading to their persistence and resulting in a set of progressive and often fatal disorders. Inhibitors have been shown to interfere with some conformations but not others, suggesting that distinct strategies must be developed to target each. However, we show here that a single dominant-negative mutant can inhibit multiple conformations of the same prion protein through the same pathway but at distinct doses. Thus, the basis of this specificity is sensitivity rather than resistance to the mechanism of inhibition, suggesting that common strategies may be used to target a range of prion conformations.

## Introduction

Alternative, self-replicating protein conformations have emerged as *bona fide* parallel protein-folding trajectories with significant biological consequences [1]. In most cases, these alternative conformations are β-sheet-rich and self-assembling, forming linear amyloid aggregates [2]. These amyloids replicate the conformation of their constituent monomers by acting as templates to direct the refolding of other conformers of the same protein as they are bound by and incorporated into the growing aggregate. In so doing, the majority of the protein is converted to the alternative conformation, changing protein activity and thereby inducing new phenotypes, such as neurodegenerative diseases (i.e., Transmissible Spongiform Encephalopathies or prion diseases, Alzheimer’s and Huntington’s diseases) and organelle biogenesis in mammals and gene expression regulation in single-celled organisms [1,3]. The high efficiency of this process, when combined with the high kinetic stability of the aggregates [2], contributes to the recalcitrance of amyloids to clearance by protein quality control pathways [4]. As a result, the associated phenotypes are frequently difficult—if not impossible—to reverse, especially in the clinic [5].

One notable exception to the persistence of amyloid-associated phenotypes is their reversal or “curing” by dominant-negative mutants of prion proteins. These sequence variants were first identified by their ability to confer resistance to scrapie in sheep (Q171R or R154H in the mammalian prion protein PrP), sporadic Creutzfeldt-Jakob disease (sCJD) in humans (E219K in PrP), and translation termination infidelity in yeast (G58D in Sup35) [6–19]. Subsequently, these mutants were shown to interfere with the assembly of amyloid by the wildtype prion protein *in vitro* and to reduce or clear existing amyloid composed of the wildtype prion protein when delivered to tissue culture cells, mice, or yeast [15,19–31]. Given this unique curing ability, elucidating the mechanism(s) by which dominant-negative prion mutants act may reveal potential strategies for reversing amyloid persistence more generally.

Despite the promise of this line of investigation, the inhibition achieved by dominant-negative mutants appears to be conformation-specific. For example, the resistance to sCJD conferred by the E219K PrP mutant in humans is not extended to the conformations, known as variants, responsible for genetic and iatrogenic forms of the disease [14,15,17,32–35]. Similarly, resistance to classical scrapie is not observed for bovine spongiform encephalopathy (BSE) or atypical scrapie variants in sheep with Q171R or R154H mutations in PrP [10,36–43] [44–52]. Finally, the G58D mutation of Sup35 cures the [*PSI*^+^]^Strong^ and [*PSI*^+^]^Sc4^ variants (*n*.*b*. [*PSI*^+^] denotes the transmissible amyloid state of Sup35) to different extents in different genetic backgrounds but is unable to cure the [*PSI*^+^]^Sc37^ and [*PSI*^+^]^Weak^ variants in yeast [53,54].

What is the molecular basis of this differential inhibition? One possibility is that the distinct recognition surfaces and/or rate-limiting steps in the self-replication process characteristic of the variants make them susceptible to only certain mechanisms of inhibition [55–61]. Alternatively, the conformational differences may confer distinct sensitivities to the same mechanism of inhibition. Given the conformational plasticity of amyloidogenic proteins [62,63], understanding the forces limiting the efficacy of inhibitors can mean the difference between developing an infinite number of individual interventions for each variant or simply different dosing regimes for the same inhibitor.

Here, we exploit the yeast prion Sup35 to gain this insight. We explored the sensitivity of three variants of Sup35 (i.e., [*PSI*^+^]^Sc4^, [*PSI*^+^]^Weak^, and [*PSI*^+^]^Sc37^) to expression of G58D and the impact of this dominant-negative mutant on the self-replication of each variant. Our studies indicate that “resistance” to G58D can be partially overcome at higher dosage of the mutant, revealing differential sensitivity to the inhibition. G58D reduces the kinetic stabilities of the amyloids associated with the variants, which determines their efficiencies of fragmentation by chaperones [60]. Consistent with this correlation, G58D inhibition of the three variants was dependent on the chaperone Hsp104, as was the case for the previously studied [*PSI*^+^]^Strong^ variant [64]. In the presence of G58D, Sup35 amyloid was fragmented by Hsp104 with higher efficiency. This increase led to amyloid clearance in daughter cells, which could be reversed by transient inhibition of Hsp104 specifically in this population. Thus, G58D dominant-negative inhibition targets distinct conformational variants through the same mechanism with differing efficacy, suggesting that the observed “resistance” is relative rather than absolute.

## Results

### [*PSI*^+^] variants are inhibited at distinct doses of G58D

To determine if the specificity of G58D on [*PSI*^+^] variants occurs through distinct mechanisms or through distinct sensitivities to the same mechanism of inhibition, we generated diploid [*PSI*^+^]^Sc4^, [*PSI*^+^]^Weak^ and [*PSI*^+^]^Sc37^ yeast strains expressing wildtype Sup35 at different ratios relative to G58D (2:1, 1:1, 1:2; S1 Fig). Inhibition of [*PSI*^+^] propagation can be monitored functionally because the formation of amyloid by Sup35 partially compromises its activity and leads to a defect in translation termination [65,66]. [*PSI*^+^] strains carrying the *ade1-14* allele form white colonies on rich medium due to read-through of a premature stop codon in the *ADE1* open reading frame. However, strains with defective prion propagation, or those that have lost the prion state (known as [*psi*^-^]), form red colonies on rich medium as a result of the accumulation of active Sup35 [67].

**Fig 1:**
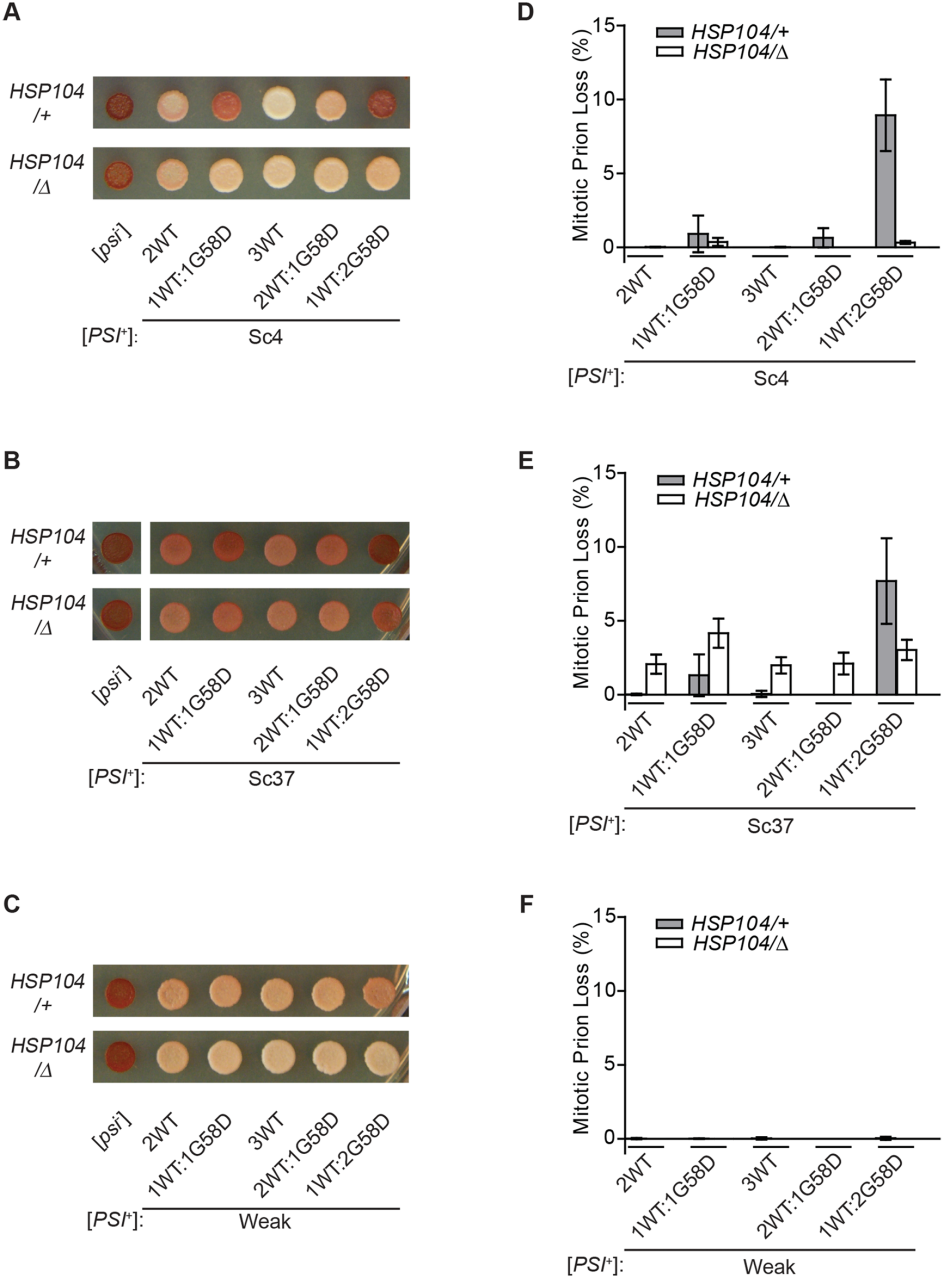
Dose-dependent effects of G58D expression on [*PSI*^+^] variants. [*PSI*^+^]^Sc4^ (*A*), [*PSI*^+^]^Sc37^ (*B*) and [*PSI*^+^]^Weak^ (*C*) wildtype (*HSP104/+*) or heterozygous-disruption (*HSP104/Δ*) diploid strains expressing wildtype (WT) and G58D Sup35 from P_SUP35_ at the indicated ratios were spotted on rich medium to analyze the [*PSI*^+^] phenotype. [*psi*^-^] diploids were included as controls. Spontaneous frequencies of [*PSI*^+^]^Sc4^ (*D*), [*PSI*^+^]^Sc37^ (*E*) and [*PSI*^+^]^Weak^ (*F*) loss during mitotic division were determined by counting the percentage of [*psi*^-^] colonies. For each strain, >3000 colonies were scored. Error bars represent standard deviations from 12 biological replicates.

Expression of G58D at any ratio in a [*PSI*^+^]^Sc4^ strain promoted the accumulation of red pigment on rich medium, indicating reversal of the prion phenotype (Fig 1A). By colony color, the severity of this effect increased with G58D dosage, with a 1:2 ratio of wildtype to G58D leading to a colony phenotype for [*PSI*^+^]^Sc4^ that was indistinguishable from [*psi*^-^] (Fig 1A). For the [*PSI*^+^]^Sc37^ and [*PSI*^+^]^Weak^ variants, which were previously reported to be compatible with G58D expression [53,54], efficient prion propagation was also dependent on the ratio of wildtype to G58D, but the critical threshold for phenotypic reversal was distinct in each case. The [*PSI*^+^]^Sc37^ variant formed colonies that were more pink on rich medium at a 1:1 ratio of wildtype to G58D relative to a wildtype strain and that were indistinguishable from [*psi*^-^] at a 1:2 ratio of wildtype to G58D (Fig 1B), mirroring our observations for [*PSI*^+^]^Sc4^ (Fig 1A). In contrast, the [*PSI*^+^]^Weak^ variant phenotype was only partially reversed at the highest ratio of wildtype to G58D tested (1:2), where the pinker colonies on rich medium relative to the wildtype [*PSI*^+^]^Weak^ strain indicated a mild inhibition by G58D (Fig 1C). Thus, the three [*PSI*^+^] variants are each dominantly inhibited by G58D expression in a dose-dependent manner, but the dose required for inhibition of [*PSI*^+^]^Sc4^ and [*PSI*^+^]^Sc37^ is lower than that of [*PSI*^+^]^Weak^.

To assess whether reversal of the [*PSI*^+^] phenotype upon G58D expression reflected prion loss (i.e., curing), we determined the frequencies of [*psi*^-^] appearance during mitotic division for each strain. [*PSI*^+^] propagation was largely stable at the 2:1 (~0% curing) and 1:1 (~1% curing) ratios of wildtype to G58D for both [*PSI*^+^]^Sc4^ and [*PSI*^+^]^Sc37^, where the colony phenotype was only mildly reversed (Fig 1A, 1B, 1D and 1E). At a 1:2 ratio of wildtype to G58D, both [*PSI*^+^]^Sc4^ (~9% curing, Fig 1D) and [*PSI*^+^]^Sc37^ (~8% curing, Fig 1E) were more unstable, consistent with the stronger reversal of their prion phenotypes at this ratio (Fig 1A and 1B). For the [*PSI*^+^]^Weak^ variant, which is less sensitive to G58D inhibition (Fig 1C), [*PSI*^+^] propagation was stable at all wildtype:G58D ratios tested (Fig 1F). Thus, [*PSI*^+^] curing in diploids expressing G58D parallels the severity of the phenotypic reversal in all three variants and, for the most sensitive strains (i.e., [*PSI*^+^]^Sc4^ and [*PSI*^+^]^Sc37^), arises in a dose-dependent manner. Together, these observations indicate that the previously described “resistance” of [*PSI*^+^]^Sc37^ and [*PSI*^+^]^Weak^ to curing by G58D expression reflected their higher threshold for sensitivity rather than their absolute recalcitrance to inhibition by this mutant.

### G58D reduces the kinetic stability of Sup35 aggregates from all [*PSI*^+^] variants

Although the three [*PSI*^+^] variants studied here, in addition to the previously studied [*PSI*^+^]^Strong^ variant [64], differ in their sensitivities to G58D inhibition (Fig 1), the dose dependence of this inhibition suggests a common underlying mechanism [64,68]. We previously linked G58D inhibition to a reduction in the kinetic stability of Sup35 aggregates and a resulting increase in their fragmentation by the chaperone Hsp104, which led to their disassembly [64]. In this model, the distinct effective inhibitory ratios of G58D on [*PSI*^+^] variants may reflect the impact that this mutant has on the kinetic stability of each. While it has been well-established that Sup35 aggregates in the [*PSI*^+^]^Sc4^ conformation are of lower stability than those in the [*PSI*^+^]^Sc37^ conformation, the relative stabilities of the four variants have not been previously reported [60,69,70].

**Fig 2:**
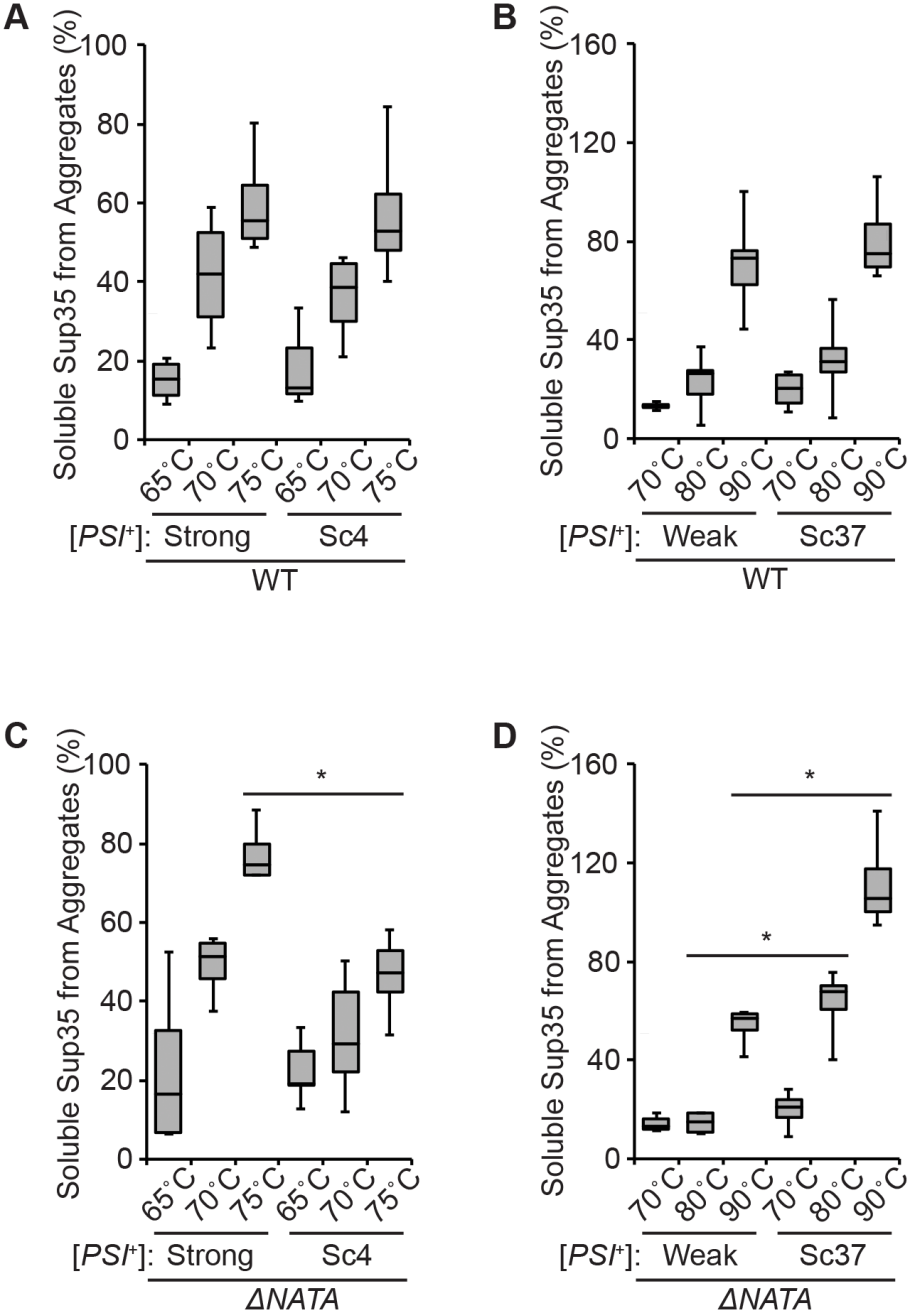
Analysis of aggregate properties for [*PSI*^+^] variants. Lysates from [*PSI*^+^]^Strong^ and [*PSI*^+^]^Sc4^ WT (*A*), [*PSI*^+^]^Weak^ and [*PSI*^+^]^Sc37^ WT (*B*), [*PSI*^+^]^Strong^ and [*PSI*^+^]^Sc4^ *ΔNATA* (*C*) or [*PSI*^+^]^Weak^ and [*PSI*^+^]^Sc37^ *ΔNATA* (*D*) haploid strains were incubated in SDS at the indicated temperatures before SDS-PAGE and quantitative immunoblotting for Sup35 (percentage of Sup35 released from aggregates at the indicated temperatures). Horizontal lines on boxes indicate 25^th^, 50^th^ and 75^th^ percentiles; whiskers indicate 10^th^ and 90^th^ percentiles. Horizontal lines indicate pair-wise comparisons (n≥4; paired t-test, *P<0.05).

To gain this insight, we first determined the kinetic stabilities of Sup35 aggregates, in the absence of G58D, by their sensitivity to disruption with 2% SDS at different temperatures as a baseline comparison [71]. Solubilized protein is then quantified by entry into a SDS-polyacrylamide gel and immunoblotting [64]. For wildtype strains, Sup35 was efficiently released from aggregates between 65°C and 75°C in lysates from strains propagating the [*PSI*^+^]^Strong^ and [*PSI*^+^]^Sc4^ variants (Fig 2A) or between 70°C and 90°C in lysates from strains propagating the [*PSI*^+^]^Weak^ and [*PSI*^+^]^Sc37^ variants (Fig 2B). The higher kinetic stability of the latter variants is consistent with their lower efficiency of fragmentation, which leads to a larger steady-state size for their associated amyloids as assessed by semi-denaturing agarose gel electrophoresis (SDD-AGE) and immunoblotting for Sup35 (S2 Fig) [60,72].

To sensitize the assay in an attempt to reveal biochemical differences between the variants in each group, we deleted the *NATA* N-terminal acetyltransferase, which reduces the kinetic stability of Sup35 amyloid in [*PSI*^+^] strains [73,74]. In this genetic background, the fraction of soluble Sup35 released from amyloid of the [*PSI*^+^]^Strong^ variant in the presence of SDS was significantly increased relative to that from the [*PSI*^+^]^Sc4^ variant over the same temperature range (Fig 2C), indicating that the aggregates are less kinetically stable in the [*PSI*^+^]^Strong^ than the [*PSI*^+^]^Sc4^ variant. Similarly, a significantly larger fraction of Sup35 was released from amyloid in the presence of SDS from the [*PSI*^+^]^Sc37^ variant than from the [*PSI*^+^]^Weak^ variant (Fig 2D), indicating that the aggregates are less kinetically stable in the [*PSI*^+^]^Sc37^ than the [*PSI*^+^]^Weak^ variant. Thus, the kinetic stability of Sup35 aggregates in [*PSI*^+^] variants increases in the order [*PSI*^+^]^Strong^, [*PSI*^+^]^Sc4^, [*PSI*^+^]^Sc37^, [*PSI*^+^]^Weak^.

**Fig 3:**
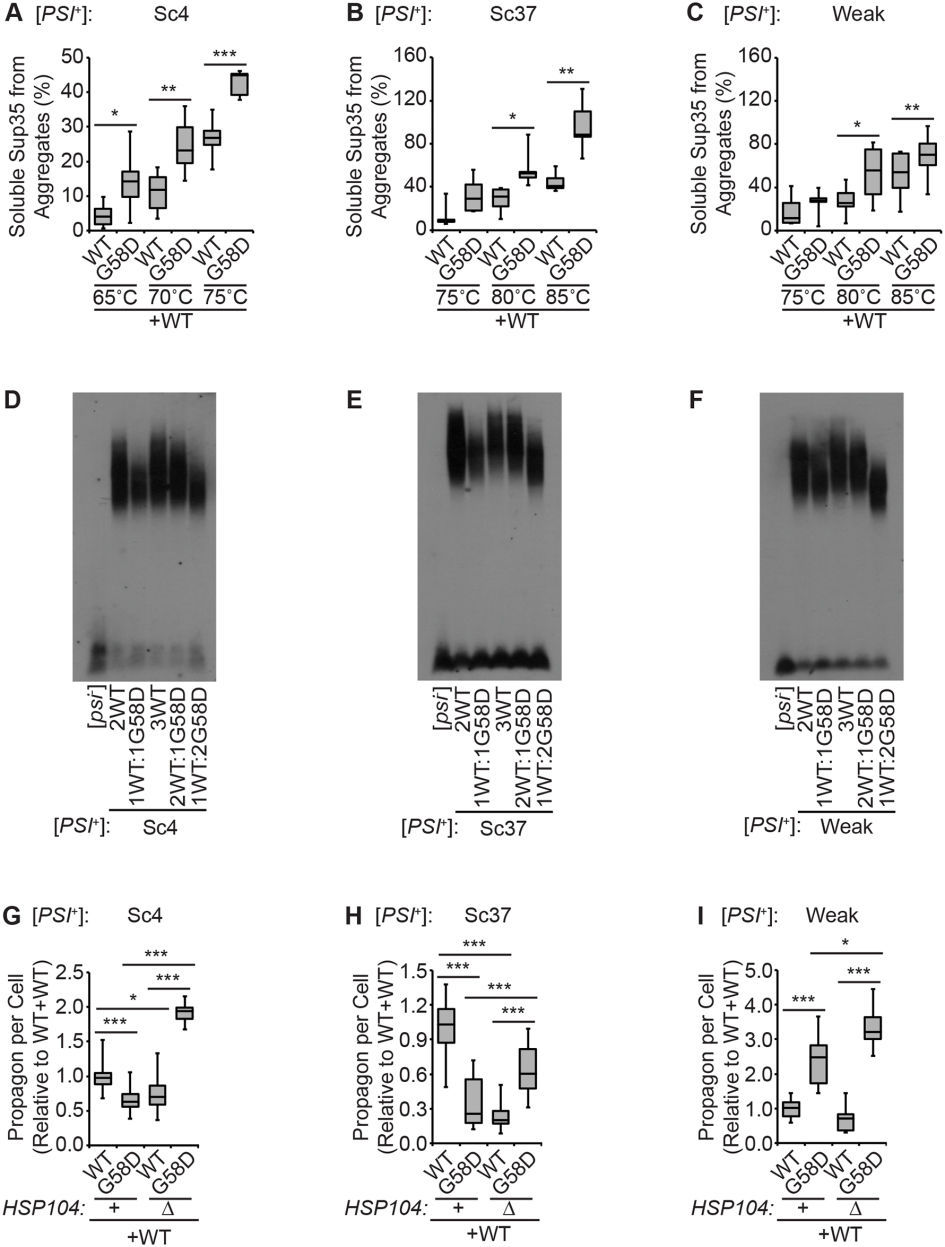
Effects of *G58D* and *HSP104* dosage on Sup35 aggregate properties. Lysates from [*PSI*^+^]^Sc4^ (*A*), [*PSI*^+^]^Sc37^ (*B*) or [*PSI*^+^]^Weak^ (*C*) diploid strains expressing one endogenous copy of *SUP35* and a second copy of *SUP35* (WT or G58D) from P_SUP35_ were incubated in SDS at the indicated temperatures before SDS-PAGE and quantitative immunoblotting for Sup35 (percentage of Sup35 released from aggregates at the indicated temperatures). Box plots are as described in the legend to Fig 2. Horizontal lines indicate pair-wise comparisons (n≥5; paired t-test, *P<0.05, **P<0.05, ***P<0.001). Lysates of [*PSI*^+^]^Sc4^ (*D*), [*PSI*^+^]^Sc37^ (*E*) or [*PSI*^+^]^Weak^ (*F*) diploid strains expressing Sup35 (WT) and G58D in the indicated ratios were analyzed by SDD-AGE and immunoblotting for Sup35. For [*PSI*^+^]^Sc4^ (*G*), [*PSI*^+^]^Sc37^ (*H*) or [*PSI*^+^]^Weak^ (*I*), the number of propagons present in individual cells was counted in wildype (+) and heterozygous *HSP104* disruption (Δ) strains, and the ratio of propagons relative to WT was determined in diploid strains expressing one endogenous copy of *SUP35* and one copy of *SUP35* (WT or G58D) from P_SUP35_. Box plots are as described in the legend to Fig 2. Horizontal lines indicate pair-wise comparisons (n≥10 cells per strain; unpaired t-test, *P<0.05, ***P<0.001).

If G58D inhibits these variants through a common mechanism, we would expect the kinetic stabilities of each of the variants to decrease in the presence of the mutant. To test this possibility, we assessed the sensitivity of Sup35 aggregates, isolated from diploid strains expressing a 1:1 ratio of wildtype to G58D, to disruption with 2% SDS at different temperatures. Soluble protein was then quantified by entry into an SDS-polyacrylamide gel and immunoblotting for Sup35. For the [*PSI*^+^]^Sc4^ strain, G58D expression increased the amount of soluble Sup35 released from aggregates at all temperatures assayed (65°C, 70°C and 75°C) in comparison with a wildtype strain (Fig 3A). G58D similarly promoted Sup35 release from aggregates isolated from the [*PSI*^+^]^Sc37^ (Fig 3B) and [*PSI*^+^]^Weak^ (Fig 3C) strains at 80°C and 85°C, but the magnitude of this effect was greater for the former. Thus, G58D incorporation destabilizes Sup35 aggregates from [*PSI*^+^] variants in a manner that correlates directly with the severity of their phenotypic inhibition (Fig 1). These observations are consistent with the idea that G58D acts through a similar mechanism to inhibit the [*PSI*^+^] variants.

### Dominant inhibition of [*PSI*^+^] variants by G58D depends on Hsp104

A decrease in the kinetic stability of amyloid should increase its efficiency of fragmentation and potentially lead to its clearance. To begin to determine the effects of G58D on the fragmentation of Sup35 amyloid associated with these [*PSI*^+^] variants, we first assessed the steady-state size distributions of these complexes by SDD-AGE and immunoblotting for Sup35. As we have previously observed for [*PSI*^+^]^Strong^ [64], expression of G58D at any ratio relative to wildtype Sup35 in a [*PSI*^+^]^Sc4^ strain led to a decrease in the accumulation of slowly migrating aggregates in comparison to the same dose of wildtype protein alone (Fig 3D). For [*PSI*^+^]^Sc37^, similar decreases were observed (Fig 3E), but for [*PSI*^+^]^Weak^, Sup35 aggregates were only shifted to smaller complexes at the lowest wildtype to G58D ratio tested (1:2, Fig 3F). Together, these observations suggest that the kinetic destabilization of Sup35 aggregates by G58D results in a higher efficiency of fragmentation *in vivo*, and these effects correlate directly with the severity of their phenotypic inhibition (Fig 1).

To determine how the kinetic destabilization of Sup35 aggregates by G58D impacts the number of heritable prion units (propagons) in [*PSI*^+^]^Sc4^, [*PSI*^+^]^Sc37^ and [*PSI*^+^]^Weak^ strains, we used a genetic assay [75]. Specifically, diploid strains expressing either two copies of wildtype *SUP35* or one copy each of wildtype *SUP35* and *G58D* were treated with guanidine HCl (GdnHCl), a potent inhibitor of the fragmentation catalyst Hsp104 [67,76–81], allowed to dilute existing aggregates through cell division, and plated in the absence of the inhibitor to quantify the number of cells inheriting an aggregate. As we have previously observed in a [*PSI*^+^]^Strong^ strain [64], G58D expression in either [*PSI*^+^]^Sc4^ and [*PSI*^+^]^Sc37^ diploids reduced propagon number by factors of ~2 and ~4, respectively (Fig 3G and 3H), consistent with the reversal of the [*PSI*^+^] phenotype and the loss of [*PSI*^+^] that we observed in these strains (Fig 1A, 1B, 1D and 1E). In contrast, G58D expression in [*PSI*^+^]^Weak^ increased propagon number by a factor of ~2.5 (Fig 3I). Although we did not detect any changes in the severity or stability of the [*PSI*^+^]^Weak^ phenotype at this ratio (Fig 1C and 1F), this increase in propagon count provides an explanation for the previously reported strengthening of the [*PSI*^+^]^Weak^ phenotype upon G58D expression to much higher levels [53]. Phenotypic strengthening is associated with a decrease in soluble Sup35, which would result from an increase in amyloid templates, detected as propagons in this assay, through enhanced fragmentation [60]. Thus, the phenotypic consequences of G58D expression, both inhibition and enhancement, can be directly explained by changes in the steady-state accumulation of Sup35 propagons. Given the distinct kinetic stabilities of Sup35 amyloid in the [*PSI*^+^] variants studied here (Fig 2), the specificity of G58D inhibition and enhancement likely reflect thresholds for fragmentation activity that result in changes in the steady-state accumulation of Sup35 forms *in vivo*.

If enhanced fragmentation is indeed the mechanism underlying G58D effects, these changes should be Hsp104-dependent. To determine if this is the case, we constructed heterozygous disruptions of *HSP104* in diploid strains expressing G58D at different ratios (S3 Fig). In strains expressing only wildtype Sup35, heterozygous disruption of *HSP104* significantly decreased the number of propagons in the [*PSI*^+^]^Sc4^ and [*PSI*^+^]^Sc37^ variants tested (Fig 3G and 3H, compare lanes 1 and 3), consistent with its catalytic role in fragmentation [78,79] and the size threshold for Sup35 aggregate transmission [72]. In contrast, heterozygous disruption of *HSP104* in [*PSI*^+^] variant strains expressing both wildtype and G58D Sup35 increased the number of propagons (Fig 3G and 3I, compare lanes 2 and 4). Thus, the reduction in propagon number associated with G58D is suppressed by lowering the dosage of *HSP104* and thereby fragmentation activity, suggesting that enhanced fragmentation is the underlying mechanism.

Next, we determined if these changes in propagon number upon heterozygous disruption of *HSP104* impacted the severity and stability of the [*PSI*^+^] phenotype. Heterozygous disruption of *HSP104* restored the [*PSI*^+^] phenotype (Fig 1A) and efficiently suppressed [*PSI*^+^] loss (Fig 1D) in the [*PSI*^+^]^Sc4^ strains expressing any ratio of G58D. For the [*PSI*^+^]^Sc37^ and [*PSI*^+^]^Weak^ variants, similar although attenuated trends were apparent. Heterozygous disruption of Hsp104 partially reversed the pinker colony color on rich medium for both [*PSI*^+^]^Sc37^ and [*PSI*^+^]^Weak^ (Fig 1B and 1C). For [*PSI*^+^]^Sc37^, heterozygous disruption of Hsp104 increased [*PSI*^+^] loss in all strains, indicating that wildtype fragmentation levels must be close to the threshold required for efficient propagation of the amyloid state (Fig 1E). Nonetheless, in the strain expressing the 1:2 ratio of wildtype to G58D, the frequency of [*PSI*^+^] loss was suppressed by heterozygous disruption of Hsp104 (Fig 1E). Thus, reduction of Hsp104 reverses the G58D-induced inhibition of the [*PSI*^+^] phenotype. Together, these observations are consistent with the idea that the downstream effect of G58D is identical for all [*PSI*^+^] variants: an enhancement of the fragmentation efficiencies of their Sup35 amyloid.

### Hsp104 mediates G58D inhibition by promoting Sup35 aggregate disassembly

The enhanced efficiency of fragmentation of Sup35 aggregates in the presence of G58D (Fig 3D and 3E) and the reduction in propagon levels (Fig 3G and 3H) suggests that Sup35 aggregates are being destroyed in strains propagating the [*PSI*^+^]^Sc4^ and [*PSI*^+^]^Sc37^ variants. For [*PSI*^+^]^Strong^, we previously detected this disassembly by monitoring the soluble pool of Sup35 in response to cycloheximide treatment to follow the fate of existing protein [64]. However, [*PSI*^+^]^Strong^ is more sensitive to G58D expression than [*PSI*^+^]^Sc4^, [*PSI*^+^]^Sc37^ and [*PSI*^+^]^Weak^ (Fig 1) [64], suggesting that release of soluble Sup35 from aggregates by enhanced fragmentation may be less readily detected in the latter variants. Specifically, the individual steps in prion propagation *in vivo* (e.g. conversion, fragmentation, and transmission) are variant-specific and difficult to monitor in isolation in a living system [60,78]. Moreover, the accumulation of soluble Sup35 is impacted not only by the inherent rate of conversion on fibers ends but also by the cumulative effect of each of the steps of prion propagation on the number of those ends [60,72]. Because the cumulative effects of each event on soluble Sup35 levels are not intuitive to qualitatively predict from those rates, we developed a deterministic model of Sup35 dynamics to deconstruct this complexity and gain additional mechanistic insight into the differential effects of G58D on the variants. This model uses a range of conversion and fragmentation rates that support [*PSI*^+^] maintenance to capture different variants (see S1 Text). In addition, we have incorporated the concept of nucleation, which specifies a minimum size for a thermodynamically stable aggregate and has been previously established as a key event in Sup35 aggregation *in vitro* [82–84].

**Fig 4:**
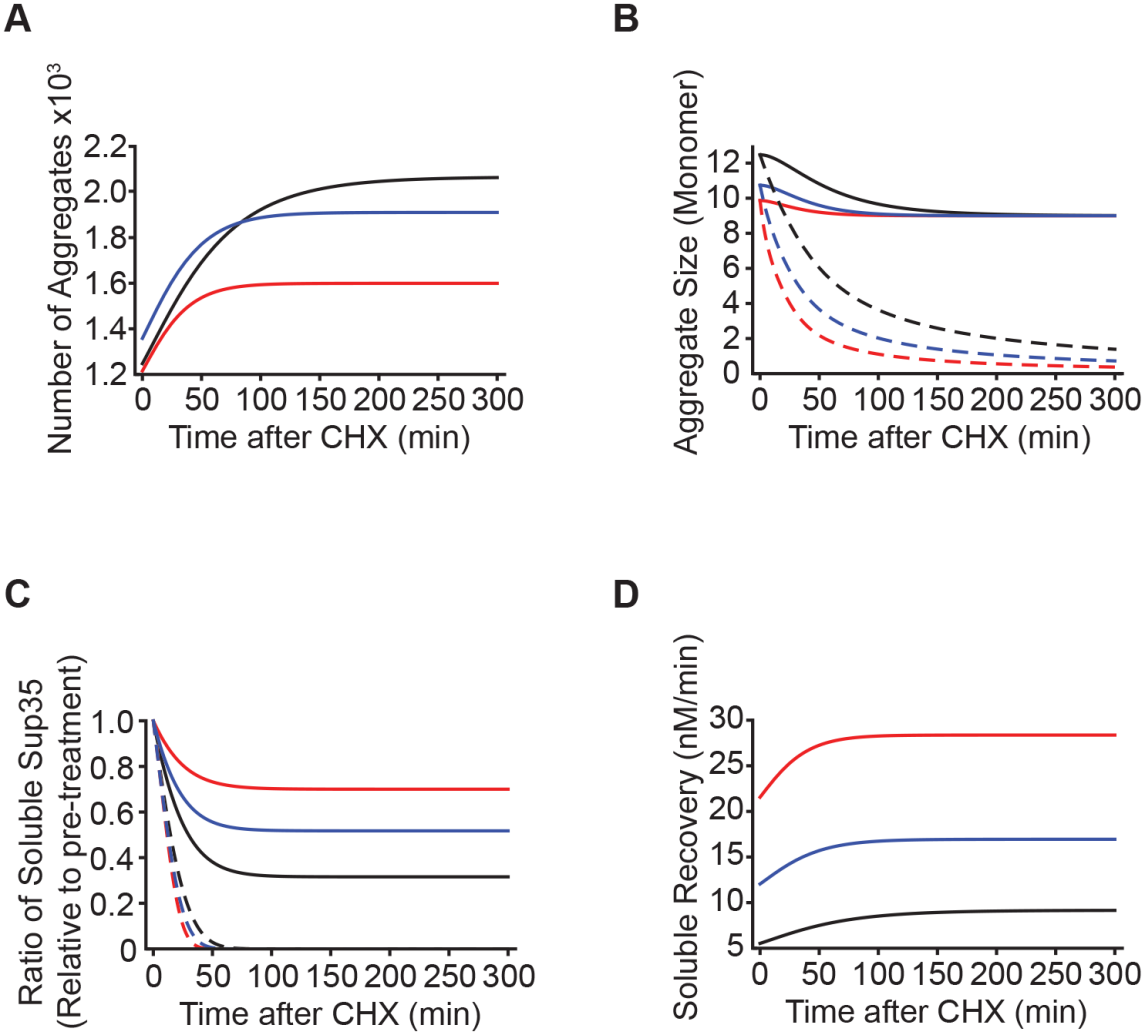
Mathematical model predicts fragmentation-dependent changes in soluble Sup35 levels in response to protein synthesis inhibition. Cycloheximide (CHX) treatment leads to an increase in the number of aggregates (*A*), an increase in average aggregate size (*B*), and a decrease in the ratio of soluble Sup35 (*C*) according to the results of stochastic simulations of a mathematical model of prion propagation. The yields (*A*, *C*) and the rate at which protein resolubilizes (*D*) vary with the rates of fragmentation: high (red), medium (blue), or low (black). The predicted changes in Sup35 average aggregate size (*B*) and soluble levels (*C*) are lost when nucleation is removed from the model (dashed lines).

The steady-state size and number of Sup35 aggregates reflects a balance between conversion, which depends on continuous synthesis of Sup35, and fragmentation [72]; when Sup35 synthesis is halted, aggregates are predicted to increase in number (Fig 4A) and decrease in size (Fig 4B) because fragmentation is proposed to exert a greater influence on the equilibrium state [72]. In line with this observation, our model predicts that cycloheximide treatment will decrease soluble Sup35 levels for prion variants that are stably propagating [*PSI*^+^] (Fig 4C) because additional templates have been created (Fig 4A).

Intriguingly, the extent of this decrease is predicted in our mathematical model to correspond inversely with the rate of fragmentation: that is, the slowest rate of fragmentation induces the largest decrease in soluble Sup35 (Fig 4B, black), relative to the steady-state levels prior to the manipulation. If fragmentation produces more templates, which in turn promotes Sup35 conversion to the amyloid state, why would we predict a lower rate of fragmentation to have the most significant effect on soluble Sup35 levels? The reason is, as we have previously demonstrated under heat shock conditions [85], fragmentation resolubilizes Sup35 in addition to creating new templates. Thus, high rates of fragmentation will push the balance between conversion and fragmentation toward the latter, causing a shift from aggregated to soluble Sup35. Consistent with this logic, our model predicts an increase in aggregate number that corresponds inversely with fragmentation rate (i.e. the largest increase in aggregate number corresponds to the slowest fragmentation rate; Fig 4A, black). This correlation can be explained directly by changes in the rate of Sup35 resolubilization from aggregates: the slowest fragmentation rate leads to the slowest rate of resolubilization (Fig 4D, black) and thereby the largest increase in aggregate number (Fig 4A, black).

**Fig 5:**
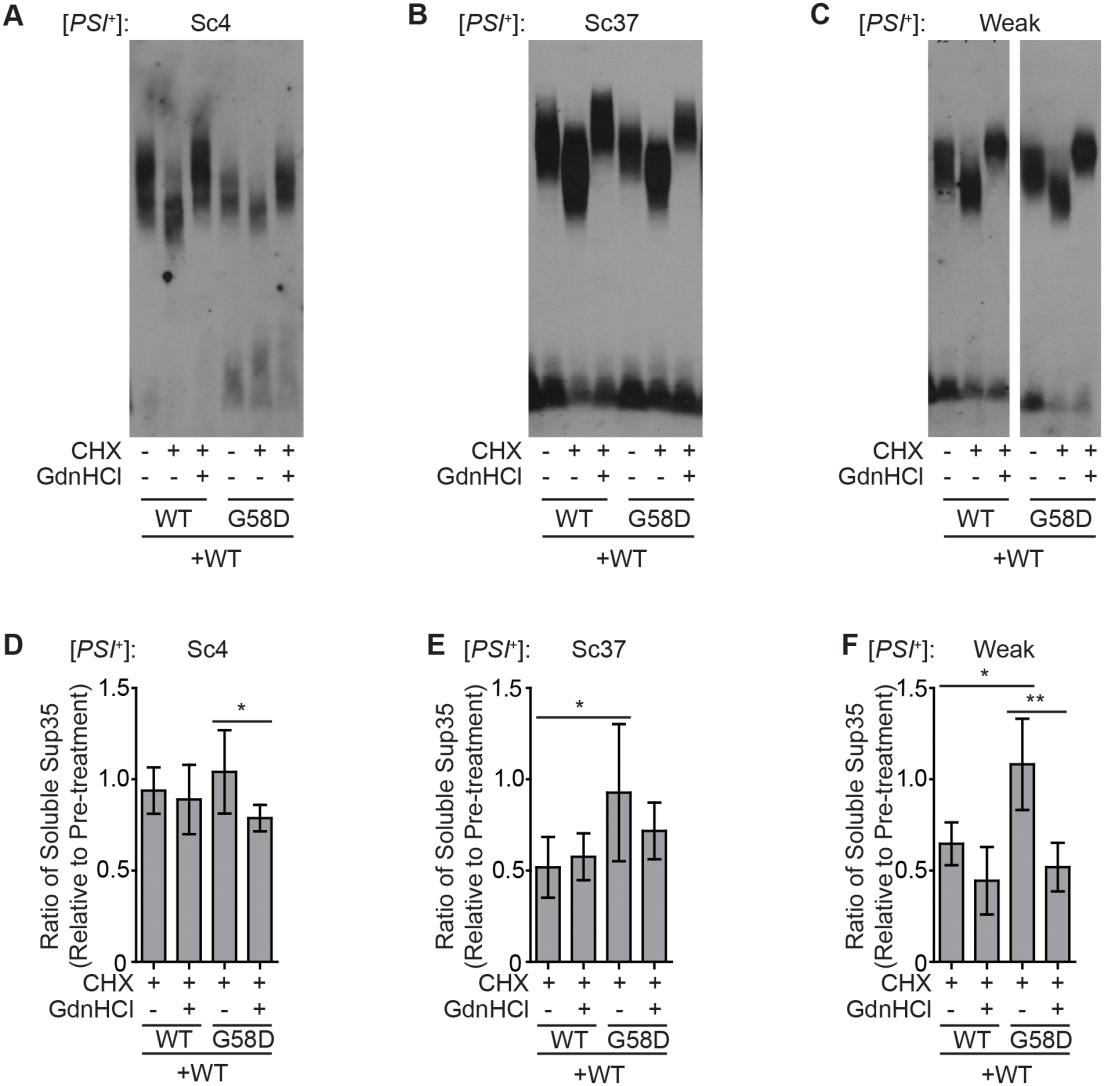
G58D expression promotes Hsp104-mediated resolubilization of aggregates. Lysates of [*PSI*^+^]^Sc4^ (*A*), [*PSI*^+^]^Sc37^ (*B*) or [*PSI*^+^]^Weak^ (*C*) strains expressing two copies of *SUP35* (WT) or one wild-type and one *G58D* copy of *SUP35* were treated with CHX or both CHX and GdnHCl and then analyzed by SDD-AGE and immunoblotting for Sup35. Lysates from diploid [*PSI*^+^]^Sc4^ (*D*), [*PSI*^+^]^Sc37^ (*E*) or [*PSI*^+^]^Weak^ (*F*) strains expressing two copies of *SUP35* (WT) or one wild-type and one *G58D* copy of *SUP35* from P_*tetO2*_ were incubated in SDS at 53°C or 100°C before SDS-PAGE and quantitative immunoblotting for Sup35, and the ratio of signal before and after treatment with CHX or both CHX and GdnHCl treatment were determined. Error bars represent standard deviations. Horizontal lines indicate pair-wise comparisons (n ≥ 5; paired t-test, *P<0.05, **P<0.01).

These predictions correlate with our observations of the [*PSI*^+^]^Sc4^, [*PSI*^+^]^Sc37^, and [*PSI*^+^]^Weak^ variants upon treatment with cycloheximide. For strains where wildtype Sup35 was the only form present, the average size of Sup35 aggregates decreased (Fig 5A–5C). In addition, the level of soluble Sup35 decreased upon cycloheximide treatment for the [*PSI*^+^]^Weak^ and [*PSI*^+^]^Sc37^ variants, but no significant decrease was observed for [*PSI*^+^]^Sc4^ variant (Fig 5D–5F, lane 1). According to our model, these observations are consistent with a nucleation-dependent aggregation process, which permits resolubilization of aggregates that are fragmented below the minimum thermodynamically stable size (Fig 4D, compare solid and dashed lines), and a higher rate of fragmentation for [*PSI*^+^]^Sc4^, which would release more aggregated Sup35 into the soluble pool (Fig 4D, red). In the presence of G58D, soluble Sup35 levels in [*PSI*^+^]^Sc37^ and [*PSI*^+^]^Weak^ are no longer reduced (Fig 5E and 5F, compare lanes 1 and 3), suggesting that G58D expression promotes aggregate fragmentation and thereby resolubilization. Consistent with this idea, treatment of the variants with both cycloheximide and guanidine HCl led to an increase in aggregate size (Fig 5A–5C) and a decrease in soluble Sup35 levels in the presence of G58D (Fig 5D–5F, compare lanes 3 and 4), indicating that Hsp104-catalyzed fragmentation promotes Sup35 resolubilization.

The ability of our mathematical model to capture the behavior of Sup35 in response to these manipulations strongly supports the idea that G58D destabilizes Sup35 aggregates to promote their increased fragmentation by Hsp104 and thereby their resolubilization. However, a more nuanced evaluation indicates that the threshold for inhibition cannot be explained by fragmentation efficiency alone. For example, [*PSI*^+^]^Sc37^ has a similar phenotypic sensitivity to G58D dosage as the [*PSI*^+^]^Sc4^ variant (Fig 1) but a kinetic stability, size, and likely fragmentation efficiency closer to the [*PSI*^+^]^Weak^ variant (Fig 2 and S2 Fig). A bulk shift in Sup35 from aggregate to soluble requires that the resolubilized Sup35 does not efficiently reconvert to the aggregated state; thus, conversion efficiencies will also impact the outcome of the G58D effects on aggregate kinetic stability, fragmentation and resolubilization. Sup35 aggregates in the [*PSI*^+^]^Sc37^ conformation direct conversion at a higher rate than those in the [*PSI*^+^]^Sc4^ conformation [60], but the relative rates of conversion for [*PSI*^+^]^Sc37^ and [*PSI*^+^]^Weak^ have not been reported. To compare these variants, we transiently treated strains with GdnHCl in liquid culture to reduce propagon number and then monitored propagon recovery as a function of time after removal of GdnHCl by plating cells and assessing their colony-color phenotype. The [*PSI*^+^]^Weak^ variant amplified its propagons at a faster rate than the [*PSI*^+^]^Sc37^ variant (S4 Fig). This recovery rate is a function of the product of the conversion and fragmentation rates [60]. Because Sup35 aggregates in the [*PSI*^+^]^Sc37^ conformation are less kinetically stable than those in the [*PSI*^+^]^Weak^ conformation (Fig 2B and 2D) and thereby likely fragmented at a higher rate, this observation suggests that the conversion rate of [*PSI*^+^]^Sc37^ is much lower than that of [*PSI*^+^]^Weak^. As a result, resolubilized Sup35 would be less likely to reconvert to the aggregated state in the [*PSI*^+^]^Sc37^ variant than in the [*PSI*^+^]^Weak^ variant. Thus, the higher rate of resolution and the lower rate of conversion combine to increase the sensitivity of [*PSI*^+^]^Sc37^ to G58D inhibition relative to [*PSI*^+^]^Weak^.

### G58D promotes Sup35 aggregate disassembly in daughter cells

**Fig 6:**
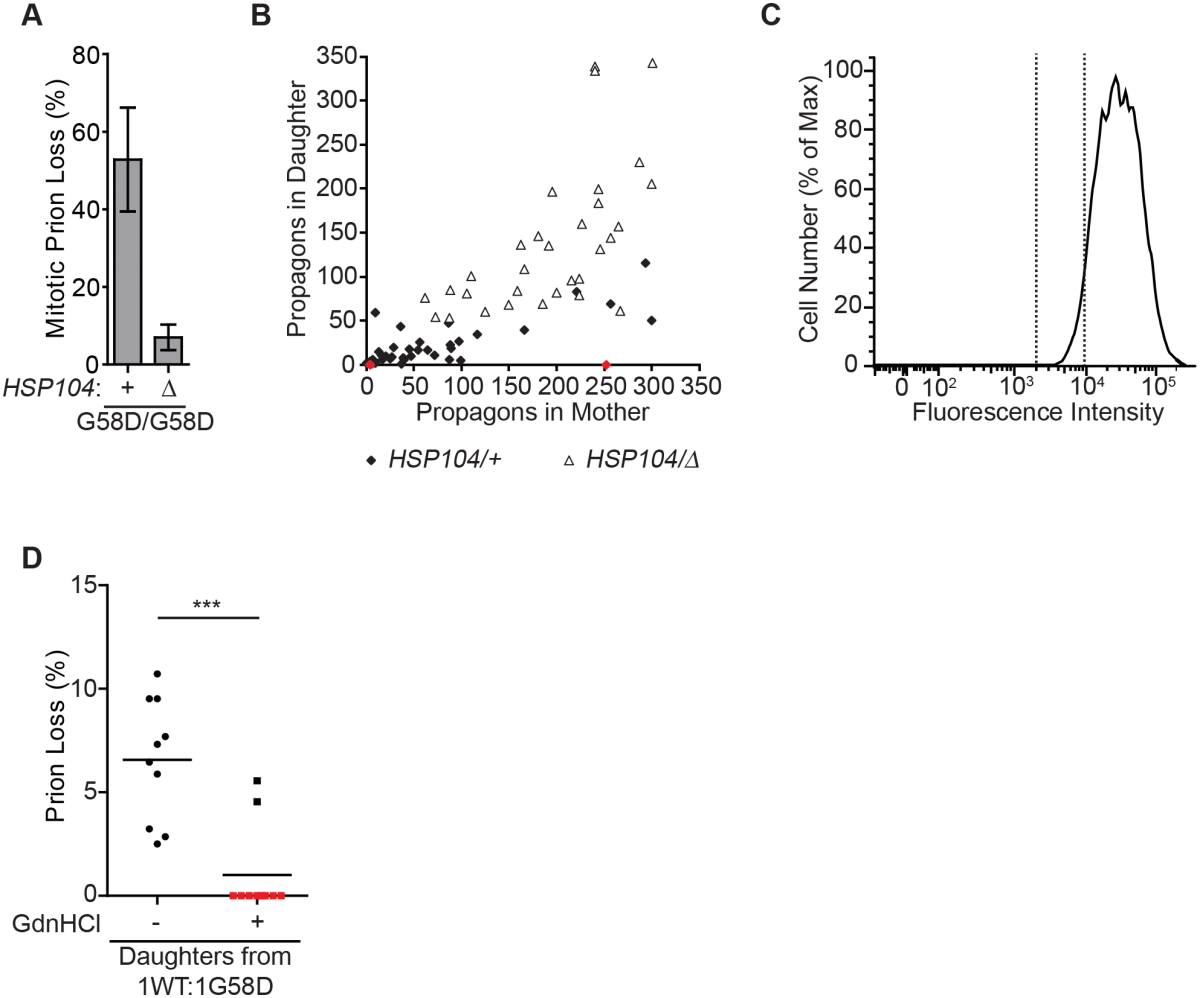
Hsp104 promotes [*PSI*^+^] curing in daughter cells expressing G58D. (*A*) Spontaneous frequencies of [*PSI*^+^]^Sc4^ loss during mitotic division were determined by counting the percentage of [*psi*^-^] colonies for strains that were wildtype (+) or heterozygous disruptions for *HSP104* (Δ). For each strain, >3000 colonies were scored. Error bars represent standard deviations from 10 biological replicates. (*B*) The number of propagons in daughter cells was plotted against the number of propagons in mother cells for wildtype (*HSP104/+*, black or red diamonds) or heterozygous *HSP104* disruption (*HSP104/Δ*, white triangles) diploid strains expressing one copy of G58D from P_SUP35_ and another copy of G58D from P_ADH_ in an [*PSI*^+^]^Sc4^ strain. Red diamonds represent mother-daughter pairs in the wildtype strain in which the mother contained propagons but the daughter did not. (*C*) The distribution of fluorescence intensities for a population of [*PSI*^+^]^Sc4^ diploid cells expressing one endogenous copy of *SUP35* and a second copy of *SUP35* (*G58D*) from P_SUP35_ stained with Alexa-647 WGA was obtained by flow cytometry. Vertical dotted lines indicate least fluorescent 5%, which was sorted as daughters. (*D*) The daughters isolated in (*C*) were plated onto minimal medium containing 3mM GdnHCl for three hours and then transferred to rich medium. The frequency of [*PSI*^+^] loss was then determined relative to that from daughters isolated from the same culture plated directly onto rich medium. Horizontal lines indicate pair-wise comparisons (n≥10 cells per strain; paired t-test, ***P<0.001).

Together, our studies are consistent with the ideas that resolubilization of aggregated Sup35 is the mechanism of G58D inhibition and that the variant-specific rates of conversion and fragmentation dictate the threshold for phenotypic reversal. However, Weissman and colleagues previously reported that loss of [*PSI*^+^]^Sc4^ propagated by G58D alone was associated with propagon loss from daughter but not mother cells [54]. This observation was interpreted as a G58D-dependent defect in Sup35 aggregate transmission to daughter cells, but using a direct fluorescence-based microscopy assay for Sup35-GFP transmission, we were unable to detect a transmission defect in [*PSI*^+^]^Strong^ strains expressing wildtype and G58D Sup35 [64]. The appearance of daughter cells without propagons could also arise if Sup35 aggregates were transmitted but subsequently disassembled by Hsp104 in this compartment. If this scenario is correct, inhibition of Hsp104 will lead to an increase in [*PSI*^+^] propagons in daughter cells. To test this hypothesis, we constructed [*PSI*^+^]^Sc4^ diploid strains expressing only G58D Sup35 and compared prion propagation in wildtype and *HSP104* heterozygous disruption versions of this strain by plating on rich medium and observing colony-color phenotype. Consistent with previous observations [54], [*PSI*^+^]^Sc4^ propagation is unstable in a wildtype strain (~50% prion loss), but we found that this instability is strongly suppressed by heterozygous disruption of *HSP104* (~5% prion loss; Fig 6A).

Propagons are normally distributed between mother and daughter cells in a 2:1 ratio [75]. However, analysis of propagon numbers in mother and daughter cells showed an even stronger bias in the distribution of propagons toward the mothers in the presence of G58D (Fig 6B, black diamonds), including a population of pairs in which the mother but not the daughter retained a large number of propagons (Fig 6B, red diamonds). By contrast, heterozygous disruption of *HSP104* reduced the stronger mother bias associated with G58D expression, and more propagons were detected in daughter cells (Fig 6B, white triangles). Notably, daughter cells lacking propagons were not isolated from the *HSP104* heterozygous disruption strain, indicating that the suppression of prion loss (Fig 6A) correlated with an increase in propagons in daughter cells (Fig 6B).

Given the suppression of these phenotypes by heterozygous disruption of Hsp104, we next directly determined if Hsp104 inhibition specifically in daughter cells is sufficient to suppress [*PSI*^+^] loss. To do so, we isolated daughter cells from [*PSI*^+^]^Sc4^ diploids expressing one copy each of wildtype and *G58D SUP35* by FACS, based on the staining of bud scars with Alexa-647 WGA. The absence of bud scars in cells with the lowest fluorescence intensity indicates that this fraction contains the newborn population, in contrast to a mixed population before sorting (Fig 6C and S5 Fig). The isolated daughters were then incubated on rich medium in the presence or absence of GdnHCl for three hours to transiently inhibit Hsp104 activity and then plated to determine the frequency of prion loss. Strikingly, GdnHCl treatment of daughter cells suppressed the frequency of prion loss (Fig 6D). Because daughter cells were biochemically isolated before treatment, the GdnHCl suppression of prion loss cannot be explained by an increased transmission of Sup35 aggregates to daughter cells upon Hsp104 inhibition. Rather, Sup35 aggregates must have already been present, with the transient inhibition of Hsp104 blocking their resolubilization after transfer, consistent with the idea that G58D inhibits the propagon of all [*PSI*^+^] variants through the same mechanism.

## Discussion

Together, our studies indicate that a single inhibitor, the dominant-negative G58D mutant of Sup35, can perturb the propagation of four different variants of the [*PSI*^+^] prion, [*PSI*^+^]^Strong^, [*PSI*^+^]^Sc4^, [*PSI*^+^]^Sc37^, and [*PSI*^+^]^Weak^, through the same mechanism. The effects of G58D expression are most easily detected at the protein level as kinetic destabilization of Sup35 amyloid (Fig 3A–3C) and related reductions in the size of their SDS-resistant core polymers (Fig 3D–3F). These changes only become apparent at the phenotypic and inheritance levels when the impact on Sup35 amyloid rises above a threshold dictated by the rates of conversion and fragmentation for the variants, allowing disassembly to dominate over reassembly.

The G58D mutation lies in the second oligopeptide repeat of Sup35, a region of the protein that is essential for prion propagation [86–88] and that impacts the ability of the Hsp104 chaperone to thread monomers through its central pore during the fragmentation process [89]. Position 58 is located within the amyloid core of Sup35 in the [*PSI*^+^]^Sc37^ variant but is more accessible in the [*PSI*^+^]^Sc4^ variant [69]. Nonetheless, the kinetic destabilization of the four variants by G58D (Fig 3A–3C) [64] suggests this region contributes directly to associations within each of the aggregates. Structural studies on the isolated second repeat revealed that the G58D substitution introduced a turn into the otherwise extended conformation of the wildtype repeat, suggesting that packing and thereby amyloid kinetic stability could be altered by this conformational change [90].

Previous studies on the [*PSI*^+^]^Strong^ and [*PSI*^+^]^Sc4^ conformational variants suggested two different mechanisms for G58D-induced curing. For [*PSI*^+^]^Strong^, curing depended not only on the dosage of *G58D* but also of *HSP104*, suggesting that prion propagation was inhibited by amyloid disassembly. Indeed, in the presence of G58D, previously aggregated Sup35 transitioned to the soluble fraction [64]. For [*PSI*^+^]^Sc4^, curing correlated with the loss of heritable aggregates in daughter cells, interpreted as a G58D-induced defect in amyloid transmission [54]. These distinct models for inhibition are consistent with the idea that different conformational variants must be cured through different molecular mechanisms [62,63]. However, our studies resolve this controversy: G58D inhibits both variants by promoting amyloid disassembly in daughter cells. This model is supported by both the Hsp104-dependence of the curing of both variants (Fig 1D) [64] and of the reduction in propagons (Fig 3H) [64]. In addition, overexpression of Hsp104 cures [*PSI*^+^]^Sc4^ propagated by G58D but not wildtype Sup35, suggesting the former is more sensitive to higher fragmentation rates than the latter [54]. Consistent with this interpretation, overexpression of an N-terminally truncated Hsp104 mutant [54], which is deficient in substrate processing [91], is unable to cure [*PSI*^+^]^Sc4^ propagated by G58D.

We have previously drawn parallels between the dominant-negative inhibition of [*PSI*^+^] propagation by Sup35 G58D and that of protease-resistant PrP by hamster Q219K (corresponding to E219K in humans and Q218K in mouse). In both cases, the mutant is incorporated into wildtype aggregates but capable of destabilizing the amyloid state only when present in excess to wildtype protein, and the efficacy of dominant-negative inhibition is greater for less kinetically stable conformational variants [15,20,21,64,92]. Given the likelihood that the mechanisms of inhibition are similar between the yeast and mammalian dominant-negative mutants, the “resistance” of sCJD to E219K in humans and of 22L to Q219K in mice may be possible to overcome by increasing the dosage of the mutant, as we have demonstrated here for G58D and [*PSI*^+^]^Sc37^ (Fig 1B and 1E). For G58D, inhibition occurs at a dosage far below that at which the prion state is induced to appear [53], indicating that the threshold between curing and induction is wide enough to accommodate switches in one direction or the other specifically. A similar analysis in mammals would be prudent before pursuing increased dosage of dominant-negative mutants as a therapeutic strategy.

How can the absence of heritable aggregates in some daughter cells be reconciled with amyloid disassembly as a common mechanism of inhibition for G58D? Our previous studies have revealed that increasing chaperone levels by heat shock, leads to amyloid disassembly in a [*PSI*^+^]^Weak^ strain [85], suggesting that the ratio of chaperones:amyloid is a key contributor to the balance between amyloid assembly and disassembly. A similar skew in this ratio likely occurs during G58D curing but through a distinct mechanism. Our previous studies uncovered a size threshold for amyloid transmission during yeast cell division: larger aggregates were preferentially retained in mother cells [72]. This asymmetry created an age-dependent difference in aggregate load, with newborn daughters taking several generations to return to the steady-state level of propagons observed in mother cells [72]. This observation suggests that the chaperone:substrate ratio could be skewed toward the former in daughter cells. This altered ratio, when combined with the decrease in the kinetic stability of Sup35 amyloid induced by G58D (Fig 3A–3C), likely creates a niche where amyloid disassembly dominates. Indeed, the normally resistant [*PSI*^+^]^Strong^ variant is cured by transient heat shock when G58D is expressed [85]. Consistent with the idea that G58D cures [*PSI*^+^] by promoting amyloid disassembly, curing is reduced (Fig 5A), and propagon numbers increase in daughters (Fig 5B) when Hsp104 levels are reduced. Most importantly, transiently blocking Hsp104 activity in daughter cells after division also greatly reduces prion loss (Fig 5D). Thus, G58D–containing Sup35 amyloid is transmitted to daughter cells, but, once there, these aggregates are at greater risk of clearance by Hsp104-mediated disassembly.

Beyond dominant-negative mutants, conformational variants of PrP and Sup35 also differ in their sensitivities to small molecule inhibitors [62,63]. Unfortunately, even sensitive conformational variants can develop resistance to these compounds, further complicating attempts to develop therapeutic interventions for these diseases. For example, treatment of prion-infected mice or tissue culture cells with quinacrine or swainsonine reduced the kinetic stability of protease-resistant PrP and altered its tropism in cell lines, but these properties were reversed when treatment was removed [93–95]. Although it remains unclear whether the emerging conformational variants were minor components that were selected or newly induced by the treatment, this conformational plasticity creates a moving target that is impossible to manage if a unique inhibitor must be developed in each case. Our studies suggest that as prion conformational variants evolve, adapt or mutate, changes in dosing regimes could be effective countermeasures, although the range of possible doses is likely to be restricted because overexpression of even a dominant-negative mutant can lead to prion appearance [53]. Nevertheless, quinacrine can eliminate the RML conformational variant of PrP from CAD5 cells at a 5-fold lower dosage than is required to eliminate an IND24-resistant variant [96].

Much research is focused on the appearance and self-replicating amplification of amyloid, yet these processes are clearly counteracted by disassembly pathways *in vivo*. This balance between assembly and disassembly contributes strongly to prion persistence, even in mammals. For example, inhibition of PrP expression can reverse accumulation of protease-resistant PrP, pathological changes and clinical progression of prion disease in mice, presumably by allowing clearance pathways to dominate, if initiated before extensive damage arises [97]. While mammals lack an Hsp104 homolog, a chaperone system, composed of mammalian Hsp70, Hsp110, and class A and B J-proteins, possesses strong disaggregase activity [98], capable of directing amyloid disassembly, although this activity has yet to be tested against protease-resistant PrP [99]. Nevertheless, natural variations in the accumulation of prion and chaperone proteins may also serve as a new framework in which to consider phenotypic differences among variants. For example, tropism and clinical progression are likely to be impacted by the balance between assembly and disassembly pathways, as we have observed for mitotic stability and heat shock-induced prion curing in yeast [72,85]. Moreover, the steady-state ratio of chaperones:amyloid may be a key consideration in screening potential therapeutics and in their ultimate efficacy *in vivo*, particularly for small molecules proteostasis regulators that perturb the assembly/disassembly balance.

## Methods

### Plasmids

All plasmids used in this study are listed in S1 Table. pRS306-P_ADH_ contains P_ADH_-Multiple Cloning Site-T_CYC1_ as a *Kpn*I-*Sac*I fragment from pSM556 (a gift from F.U. Hartl) in a similarly digested pRS306. The *SUP35(G58D*) ORF was then subcloned into pRS306-P_ADH_ as a *Bam*HI-*Eco*RI fragment isolated from pRS306-SUP35(G58D) to create pRS306-P_ADH_SUP35(G58D) (SB468).

### Oligonucleotides

Oligonucleotides used in this study are listed in S2 Table.

### Yeast strains

All strains are derivatives of 74-D694 and are listed in S3 Table. [*PSI*^+^]^Sc4^ (SY2085) and [*PSI*^+^]^Sc37^ (SY2086) haploid wildtype strains were gifts from J. Weissman. Yeast strains expressing ectopic copies of *SUP35* or *G58D* from *URA3* (pRS306) or *TRP1* (pRS304)-marked plasmids were constructed by transforming yeast strains with plasmids that were linearized with *Bst*BI or *Bsu*361, respectively, and by selecting for transformants on the appropriate minimal medium. In all cases, expression was confirmed by quantitative immunoblotting for Sup35. Disruptions of *SUP35* (FP35, FP36) were generated by transformation of PCR-generated cassettes using pFA6aKanMX4 as a template with the indicated oligonucleotide primers (S2 Table) and selection on rich medium supplemented with G418. *HSP104* disruptions were generated by transformation with a *Pvu*I-*Bam*HI fragment of pYABL5 (a gift from S. Lindquist) and selection on minimal medium lacking leucine. Disruptions of *NAT1* (FP29, FP30) were generated by transformation of PCR-generated cassettes using pFA6a-hphMX4 as a template with the indicated primers (S2 Table) and selection on complete medium supplemented with hygromycin. All the disruptions were verified by PCR and 2:2 segregation of the appropriate marker.

### Prion loss

Exponentially growing cultures of the indicated strain were plated on YPD for single colonies, and the frequency of [*PSI*^+^] loss was determined by the number of red colonies arising.

### Protein analysis

Semidenaturing detergent agarose gel electrophoresis (SDD-AGE), SDS-PAGE, quantitative immunoblotting and SDS-sensitivity experiments were performed as previously described [73]. To analyze the fate of aggregated Sup35, cultures were grown to midlog phase and treated with cycloheximide (CHX) or both CHX and guanidine HCl (GdnHCl) for 1.7 hours. Yeast lysates were collected before and after treatment and incubated at 53°C and 100°C in the presence of 2% SDS before analysis by SDS-PAGE. Lysates were also prepared from the same cultures and analyzed by SDD-AGE.

### Propagon counts

The number of propagons per cell was determined using a previously described *in vivo* dilution, colony-based method [75]. For propagon counting in mothers and daughters, a pair of mother and daughter cells was separated by micromanipulation onto minimal medium (SD-complete with 2.5mM adenine and 4% dextrose) with 3mM GdnHCl. After growing at 30°C for about 48 h, whole colonies were isolated using a cut pipette tip, resuspended in a small volume of water and plated onto YPD plates. The number of white colonies was then counted.

### Daughter-specific assays

Daughters were separated by FACS based on bud-scar labeling. Yeast cells were incubated for 1 h at room temperature in 1μg/ml Alexa-647 wheat germ agglutinin (WGA) in PBS. After washing twice in PBS, cells with the lowest fluorescence intensity (5%) were sorted as newborn daughter cells, and a sample of this fraction was viewed by fluorescence microscopy to confirm bud scar number. This fraction was also moved to rich medium (1/4 YPD) for color development. For Hsp104 inhibition, sorted fractions were first moved to a minimal medium with 3mM GdnHCl for three hours before being transferred to rich medium. In each case only completely red colonies were counted as [*psi*^-^].

### Fluorescence microscopy

Fluorescence microscopy was performed on a DeltaVision deconvolution microscope equipped with a 100x objective. WGA Alexa-647 fluorescence was collected using 650nm excitation and 668nm emission wavelengths and with an exposure of 50ms. Images were processed in ImageJ software.

### Propagon recovery

Cultures were grown in YPAD medium to an OD_600_ of 0.1 at 30°C. GdnHCl was added to 3mM, and the culture was returned to 30°C for 5 hours to decrease the propagon number. Cultures were then collected by centrifugation, washed and transferred to YPAD medium without GdnHCl for recovery. Samples were plated on YPD, and the number of propagons per cell was counted at the indicated timepoints.

## Supporting information

S1 Fig Sup35 expression level is consistent with copy number. Lysates isolated from [*PSI*^+^]^Sc4^ (*A*), [*PSI*^+^]^Sc37^ (*B*) or [*PSI*^+^]^Weak^ (*C*) strains described in Fig 1A–1C were analyzed by SDS-PAGE and quantitative immunoblotting for Sup35. The ratio of Sup35 relative to a wildtype diploid is shown. Error bars represent standard deviation.
(TIF)

S2 Fig [*PSI*^+^] variants differ in the size of their Sup35 aggregates. Lysates from the indicated [*PSI*^+^] variant haploid yeast strains were analyzed by SDD-AGE and immunoblotting for Sup35.
(TIF)

S3 Fig Hsp104 expression level is consistent with copy number. Lysates isolated from [*PSI*^+^]^Sc4^ (*A*), [*PSI*^+^]^Sc37^ (*B*) or [*PSI*^+^]^Weak^ (*C*) strains described in Fig 1A–1C were analyzed by SDS-PAGE and quantitative immunoblotting for Hsp104. The ratio of Hsp104 relative to a wild-type diploid is shown. Error bars represent standard deviation.
(TIF)

S4 Fig [*PSI*^+^]^Sc37^ and [*PSI*^+^]^Weak^ recover propagons at different rates. The rate of propagon recovery was determined for [*PSI*^+^]^Sc37^ (blue) and [*PSI*^+^]^Weak^ (red) after treatment with GdnHCl. Error bars represent standard deviation. (n ≥ 12 cells per strain per time point; unpaired t-test, **P<0.01).
(TIF)

S5 Fig Analysis of FACS-sorted daughter cells. DIC and Alexa-647 WGA fluorescence of bud scars of cells both before and after FACS sorting. Scale bars represent 3μm.
(TIF)

S1 Table Plasmids. (DOCX)

S2 Table Oligonucleotide sequences. (DOCX)

S3 Table Yeast strains. (DOCX)

S1 Text Mathematical model. (PDF)
