# Supplementary material for: A dominant-negative mutant inhibits multiple prion variants through a common mechanism
Source: PLoS Genet. 2017 Oct 30;13(10):e1007085. doi: 10.1371/journal.pgen.1007085 (PMC5679637; doi:10.1371/journal.pgen.1007085)
Supplement: S1 Table — (DOCX) [file pgen.1007085.s006.docx]

**S1 Table: Plasmids**

| **Name** | **Description** | **Reference** |
| --- | --- | --- |
| 6686 | pRS306-P_SUP35_SUP35 | DiSalvo *et al.* 2011 |
| SB467 | pRS306-P_SUP35_SUP35(G58D) | DiSalvo *et al.* 2011 |
| SB468 | pRS306-P_ADH_SUP35(G58D) | This study |
| SB645 | pRS304-P_SUP35_SUP35(G58D) | DiSalvo *et al.* 2011 |
| SB657 | pRS306-P_tet02_SUP35 | DiSalvo *et al.* 2011 |
| SB658 | pRS306-P_tet02_SUP35(G58D) | DiSalvo *et al.* 2011 |
