# Supplementary material for: A dominant-negative mutant inhibits multiple prion variants through a common mechanism
Source: PLoS Genet. 2017 Oct 30;13(10):e1007085. doi: 10.1371/journal.pgen.1007085 (PMC5679637; doi:10.1371/journal.pgen.1007085)
Supplement: S2 Table — (DOCX) [file pgen.1007085.s007.docx]

**S2 Table: Oligonucleotide Sequences**

| **Name** | **Description** | **Sequence (5’-3’)** |
| --- | --- | --- |
| FP29 | 5’NAT1 KO | GACAAATACCATTGAGGAAGGCGATTGACCCTAACGAAGTCAGCTGAAGCTTCGTACGC |
| FP30 | 3’NAT1 KO | AATTAAGTAAGAGTTAATTGACACATTGAGGAGTTGCAGGGCATAGGCCACTAGTGGATCTG |
| FP31 | 5’NAT1 KO CHK | AAGCAGTAGGAAAATTGGTGTGG |
| FP32 | 3’NAT1 KO CHK | CTGATCGCGTCTTTATCTTGTG |
| FP33 | PTEF CHK | GCACGTCAAGACTGTCAAGG |
| FP34 | pFA6a | TGCCCAGATGCGAAGTTAAGTG |
| FP35 | 5’SUP35 KO | ACTTGCTCGGAATAACATCTATATCTGCCCACTAGCAACACGGATCCCCGGGTTAATTAA |
| FP36 | 3’SUP35 KO | GGTATTATTGTGTTTGCATTTACTTATGTTTGCAAGAAATGAATTCGAGCTCGTTTAAAC |
| FP75 | 5'HSP104 promoter | CCATCGATTCAAAGGCGTTATT |
| FP76 | 3'HSP104 | TCATACTTTGGTTGCAGAC |
| FP77 | 5'LEU2 test | CACATGAACAAGGAAGTACAG |
| FP78 | 3'LEU2 test | AGAAACGGCCTTAACGAC |
| FP93 | 5'SUP35 KO CHK | CACAAAAATCATACAACGAATGG |
| FP108 | 3’SUP35 KO CHK | CTAAATGATGTTGACAAACTTATG |
