# Supplementary material for: A dominant-negative mutant inhibits multiple prion variants through a common mechanism
Source: PLoS Genet. 2017 Oct 30;13(10):e1007085. doi: 10.1371/journal.pgen.1007085 (PMC5679637; doi:10.1371/journal.pgen.1007085)
Supplement: S3 Table — (DOCX) [file pgen.1007085.s008.docx]

**S3 Table: Yeast Strains**

| **Strains** | **Genotype** | **Plasmids Integrated** | **Figure** | **Reference** |
| --- | --- | --- | --- | --- |
| SLL2606 | *MATa* [*PSI*^+^]^Strong^ *ade1-14 his3Δ200 trp1-289 ura3-52 leu2-3, 112* | - | 2A, S2 | Chernoff et al. 1995 |
| SLL2600 | *MATa* [*PSI*^+^]^Weak^ *ade1-14 his3Δ200 trp1-289 ura3-52 leu2-3, 112* | - | 2B, S2, S4 | Derkatch et al. 1996 |
| SLL3261 | *MATa/α* [*psi*^-^] *ade1-14/ade1-14 his3Δ200/his3Δ200 trp1-289/ trp1-289 ura3-52/ura3-52 leu2-3, 112/ leu2-3, 112* | - | 1A, 1B, 1C, 3D, 3E, 3F | DiSalvo et al. 2011 |
| SY320 | *MATα* [*PSI*^+^]^Strong^ *ade1-14 his3Δ200 trp1-289 ura3-52 leu2-3, 112 nata::hphMX4* | - | 2C | This study |
| SY1773 | *MATa/α* [*PSI*^+^]^Weak^ *ade1-14/ade1-14 his3Δ200/his3Δ200 trp1-289/ trp1-289 ura3-52/ura3-52::URA3::P_SUP35_SUP35 leu2-3, 112/ leu2-3, 112* | 6686 | 1C, 1F, 3F, S1C | This study |
| SY1774 | *MATa/α* [*PSI*^+^]^Weak^ *ade1-14/ade1-14 his3Δ200/his3Δ200 trp1-289/ trp1-289 ura3-52/ura3-52::URA3::P_SUP35_SUP35(G58D) leu2-3, 112/ leu2-3, 112* | SB467 | 1C, 1F, 3F, S1C | This study |
| SY1776 | *MATa/α* [*PSI*^+^]^Weak^ *ade1-14/ade1-14 his3Δ200/his3Δ200 trp1-289/ trp1-289 ura3-52/ura3-52::URA3::P_SUP35_SUP35 leu2-3, 112/ leu2-3, 112 SUP35/sup35::kanMX4* | 6686 | 1C, 1F, 3C, 3F, 3I, S1C | This study |
| SY1777 | *MATa/α* [*PSI*^+^]^Weak^ *ade1-14/ade1-14 his3Δ200/his3Δ200 trp1-289/ trp1-289 ura3-52/ura3-52::URA3::P_SUP35_SUP35(G58D) leu2-3, 112/ leu2-3, 112 SUP35/sup35::kanMX4* | SB467 | 1C, 1F, 3C, 3F, 3I, S1C | This study |
| SY1780 | *MATa/α* [*PSI*^+^]^Weak^ *ade1-14/ade1-14 his3Δ200/his3Δ200 trp1-289/ trp1-289::TRP1:: P_SUP35_SUP35(G58D) ura3-52/ura3-52::URA3::P_SUP35_SUP35(G58D) leu2-3, 112/ leu2-3, 112 SUP35/sup35::kanMX6* | SB467, SB645 | 1C, 1F, 3F, S1C | This study |
| SY2085 | *MATa* [*PSI*^+^]^Sc4^ *ade1-14 his3Δ200 trp1-289 ura3-52 leu2-3, 112* | - | 2A, S2 | Tanaka et al. 2006 |
| SY2086 | *MATa* [*PSI*^+^]^Sc37^ *ade1-14 his3Δ200 trp1-289 ura3-52 leu2-3, 112* | - | 2B, S2, S4 | Tanaka et al. 2006 |
| SY2248 | *MATα* [*PSI*^+^]^Sc4^ *ade1-14 his3Δ200 trp1-289 ura3-52 leu2-3, 112 nata::LEU2* | - | 2C | This study |
| SY2257 | *MATa/α* [*PSI*^+^]^Sc4^ *ade1-14/ade1-14 his3Δ200/his3Δ200 trp1-289/ trp1-289 ura3-52/ura3-52::URA3::P_SUP35_SUP35 leu2-3, 112/ leu2-3, 112* | 6686 | 1A, 1D, 3D, S1A | This study |
| SY2258 | *MATa/α* [*PSI*^+^]^Sc4^ *ade1-14/ade1-14 his3Δ200/his3Δ200 trp1-289/ trp1-289 ura3-52/ura3-52 leu2-3, 112/ leu2-3, 112* | - | 1A, 1D, 3A, 3D, 3G, S1A | This study |
| SY2259 | *MATa/α* [*PSI*^+^]^Sc4^ *ade1-14/ade1-14 his3Δ200/his3Δ200 trp1-289/ trp1-289 ura3-52/ura3-52::URA3::P_SUP35_SUP35(G58D) leu2-3, 112/ leu2-3, 112 SUP35/sup35::kanMX4* | SB467 | 1A, 1D, 3A, 3D, 3G, 6C, 6D, S1A, S5 | This study |
| SY2260 | *MATa/α* [*PSI*^+^]^Sc4^ *ade1-14/ade1-14 his3Δ200/his3Δ200 trp1-289/ trp1-289::TRP1:: P_SUP35_SUP35(G58D) ura3-52/ura3-52::URA3::P_SUP35_SUP35(G58D) leu2-3, 112/ leu2-3, 112 SUP35/sup35::kanMX6* | SB467, SB645 | 1A, 1D, 3D, S1A | This study |
| SY2261 | *MATa/α* [*PSI*^+^]^Sc4^ *ade1-14/ade1-14 his3Δ200/his3Δ200 trp1-289/ trp1-289 ura3-52/ura3-52::URA3::P_SUP35_SUP35(G58D) leu2-3, 112/ leu2-3, 112* | SB467 | 1A, 1D, 3D, S1A | This study |
| SY2281 | *MATa/α* [*PSI*^+^]^Sc4^ *ade1-14/ade1-14 his3Δ200/his3Δ200 trp1-289/ trp1-289 ura3-52/ura3-52::URA3::P_SUP35_SUP35 leu2-3, 112/ leu2-3, 112 HSP104/hsp104::LEU2* | 6686 | 1A, 1D, S3A | This study |
| SY2283 | *MATa/α* [*PSI*^+^]^Sc4^ *ade1-14/ade1-14 his3Δ200/his3Δ200 trp1-289/ trp1-289 ura3-52/ura3-52 leu2-3, 112/ leu2-3, 112 HSP104/hsp104::LEU2* | - | 1A, 1D, 3G, S3A | This study |
| SY2285 | *MATa/α* [*PSI*^+^]^Sc4^ *ade1-14/ade1-14 his3Δ200/his3Δ200 trp1-289/ trp1-289 ura3-52/ura3-52::URA3::P_SUP35_SUP35(G58D) leu2-3, 112/ leu2-3, 112 HSP104/hsp104::LEU2* | SB467 | 1A, 1D, S3A | This study |
| SY2287 | *MATa/α* [*PSI*^+^]^Sc4^ *ade1-14/ade1-14 his3Δ200/his3Δ200 trp1-289/ trp1-289 ura3-52/ura3-52::URA3::P_SUP35_SUP35(G58D) leu2-3, 112/ leu2-3, 112 SUP35/sup35::kanMX4 HSP104/hsp104::LEU2* | SB467 | 1A, 1D, 3G, S3A | This study |
| SY2289 | *MATa/α* [*PSI*^+^]^Sc4^ *ade1-14/ade1-14 his3Δ200/his3Δ200 trp1-289/ trp1-289::TRP1:: P_SUP35_SUP35(G58D) ura3-52/ura3-52::URA3::P_SUP35_SUP35(G58D) leu2-3, 112/ leu2-3, 112 SUP35/sup35::kanMX6 HSP104/hsp104::LEU2* | SB467, SB645 | 1A, 1D, 3G, S3A | This study |
| SY2535 | *MATa/α* [*PSI*^+^]^Weak^ *ade1-14/ade1-14 his3Δ200/his3Δ200 trp1-289/ trp1-289 ura3-52/ura3-52::URA3::P_SUP35_SUP35 leu2-3, 112/ leu2-3, 112 HSP104/hsp104::LEU2* | 6686 | 1C, 1F, S3C | This study |
| SY2536 | *MATa/α* [*PSI*^+^]^Weak^ *ade1-14/ade1-14 his3Δ200/his3Δ200 trp1-289/ trp1-289 ura3-52/ura3-52 leu2-3, 112/ leu2-3, 112 HSP104/hsp104::LEU2* | - | 1C, 1F, 3I, S3C | This study |
| SY2537 | *MATa/α* [*PSI*^+^]^Weak^ *ade1-14/ade1-14 his3Δ200/his3Δ200 trp1-289/ trp1-289 ura3-52/ura3-52::URA3::P_SUP35_SUP35(G58D) leu2-3, 112/ leu2-3, 112 HSP104/hsp104::LEU2* | SB467 | 1C, 1F, S3C | This study |
| SY2538 | *MATa/α* [*PSI*^+^]^Weak^ *ade1-14/ade1-14 his3Δ200/his3Δ200 trp1-289/ trp1-289 ura3-52/ura3-52::URA3::P_SUP35_SUP35(G58D) leu2-3, 112/ leu2-3, 112 SUP35/sup35::kanMX4 HSP104/hsp104::LEU2* | SB467 | 1C, 1F, 3I, S3C | This study |
| SY2539 | *MATa/α* [*PSI*^+^]^Weak^ *ade1-14/ade1-14 his3Δ200/his3Δ200 trp1-289/ trp1-289::TRP1:: P_SUP35_SUP35(G58D) ura3-52/ura3-52::URA3::P_SUP35_SUP35(G58D) leu2-3, 112/ leu2-3, 112 SUP35/sup35::kanMX6 HSP104/hsp104::LEU2* | SB467, SB645 | 1C, 1F, S3C | This study |
| SY2812 | *MATα* [*PSI*^+^]^Sc37^ *ade1-14 his3Δ200 trp1-289 ura3-52 leu2-3, 112 nata::hphMX4* | - | 2D | This study |
| SY2847 | *MATa/α* [*PSI*^+^]^Sc37^ *ade1-14/ade1-14 his3Δ200/his3Δ200 trp1-289/ trp1-289 ura3-52/ura3-52::URA3::P_SUP35_SUP35 leu2-3, 112/ leu2-3, 112* | 6686 | 1C, 1E, 3E, S1B | This study |
| SY2848 | *MATa/α* [*PSI*^+^]^Sc37^ *ade1-14/ade1-14 his3Δ200/his3Δ200 trp1-289/ trp1-289 ura3-52/ura3-52 leu2-3, 112/ leu2-3, 112* | - | 1B, 1E, 3B, 3E, 3H, 5B, 5E, S1B | This study |
| SY2849 | *MATa/α* [*PSI*^+^]^Sc37^ *ade1-14/ade1-14 his3Δ200/his3Δ200 trp1-289/ trp1-289 ura3-52/ura3-52::URA3::P_SUP35_SUP35(G58D) leu2-3, 112/ leu2-3, 112* | SB467 | 1B, 1E, 3E, S1B | This study |
| SY2850 | *MATa/α* [*PSI*^+^]^Sc37^ *ade1-14/ade1-14 his3Δ200/his3Δ200 trp1-289/ trp1-289 ura3-52/ura3-52::URA3::P_SUP35_SUP35(G58D) leu2-3, 112/ leu2-3, 112 SUP35/sup35::kanMX4* | SB467 | 1B, 1E, 3B, 3E, 3H, 5B, 5E, S1B | This study |
| SY2851 | *MATa/α* [*PSI*^+^]^Sc37^ *ade1-14/ade1-14 his3Δ200/his3Δ200 trp1-289/ trp1-289::TRP1:: P_SUP35_SUP35(G58D) ura3-52/ura3-52::URA3::P_SUP35_SUP35(G58D) leu2-3, 112/ leu2-3, 112 SUP35/sup35::kanMX6* | SB467, SB645 | 1B, 1E, 3E, S1B | This study |
| SY2856 | *MATa/α* [*PSI*^+^]^Sc37^ *ade1-14/ade1-14 his3Δ200/his3Δ200 trp1-289/ trp1-289 ura3-52/ura3-52::URA3::P_SUP35_SUP35 leu2-3, 112/ leu2-3, 112 HSP104/hsp104::LEU2* | 6686 | 1B, 1E, S3B | This study |
| SY2857 | *MATa/α* [*PSI*^+^]^Sc37^ *ade1-14/ade1-14 his3Δ200/his3Δ200 trp1-289/ trp1-289 ura3-52/ura3-52 leu2-3, 112/ leu2-3, 112 HSP104/hsp104::LEU2* | - | 1B, 1E, 3H, S3B | This study |
| SY2858 | *MATa/α* [*PSI*^+^]^Sc37^ *ade1-14/ade1-14 his3Δ200/his3Δ200 trp1-289/ trp1-289 ura3-52/ura3-52::URA3::P_SUP35_SUP35(G58D) leu2-3, 112/ leu2-3, 112 HSP104/hsp104::LEU2* | SB467 | 1B, 1E, S3B | This study |
| SY2859 | *MATa/α* [*PSI*^+^]^Sc37^ *ade1-14/ade1-14 his3Δ200/his3Δ200 trp1-289/ trp1-289 ura3-52/ura3-52::URA3::P_SUP35_SUP35(G58D) leu2-3, 112/ leu2-3, 112 SUP35/sup35::kanMX4 HSP104/hsp104::LEU2* | SB467 | 1B, 1E, 3H, S3B | This study |
| SY2860 | *MATa/α* [*PSI*^+^]^Sc37^ *ade1-14/ade1-14 his3Δ200/his3Δ200 trp1-289/ trp1-289::TRP1:: P_SUP35_SUP35(G58D) ura3-52/ura3-52::URA3::P_SUP35_SUP35(G58D) leu2-3, 112/ leu2-3, 112 SUP35/sup35::kanMX6 HSP104/hsp104::LEU2* | SB467, SB645 | 1B, 1E, S3B | This study |
| SY2862 | *MATα* [*PSI*^+^]^Weak^ *ade1-14 his3Δ200 trp1-289 ura3-52 leu2-3, 112 nata::hphMX4* | - | 2D | This study |
| SY2878 | *MATa/α* [*PSI*^+^]^Weak^ *ade1-14/ade1-14 his3Δ200/his3Δ200 trp1-289/ trp1-289 ura3-52/ura3-52::URA3::P_tet02_SUP35 leu2-3, 112/ leu2-3, 112 SUP35/sup35::kanMX4* | SB657 | 5C, 5F | This study |
| SY2879 | *MATa/α* [*PSI*^+^]^Weak^ *ade1-14/ade1-14 his3Δ200/his3Δ200 trp1-289/ trp1-289 ura3-52/ura3-52::URA3::P_tet02_SUP35(G58D) leu2-3, 112/ leu2-3, 112 SUP35/sup35::kanMX4* | SB658 | 5C, 5F | This study |
| SY2957 | *MATa/α* [*PSI*^+^]^Sc4^ *ade1-14/ade1-14 his3Δ200/his3Δ200 trp1-289/ trp1-289 ura3-52::URA3::P_ADH_SUP35(G58D)/ura3-52::URA3::P_SUP35_SUP35(G58D) leu2-3, 112/ leu2-3, 112 sup35::kanMX4/sup35::kanMX4* | SB467， SB468 | 6A, 6B | This study |
| SY2976 | *MATa/α* [*PSI*^+^]^Sc4^ *ade1-14/ade1-14 his3Δ200/his3Δ200 trp1-289/ trp1-289 ura3-52::URA3::P_ADH_SUP35(G58D)/ura3-52::URA3::P_SUP35_SUP35(G58D) leu2-3, 112/ leu2-3, 112 sup35::kanMX4/sup35::kanMX4 HSP104/hsp104::LEU2* | SB467， SB468 | 6A, 6B | This study |
| SY3044 | *MATa/α* [*PSI*^+^]^Sc4^ *ade1-14/ade1-14 his3Δ200/his3Δ200 trp1-289/ trp1-289 ura3-52/ura3-52::URA3::P_tet02_SUP35 leu2-3, 112/ leu2-3, 112 SUP35/sup35::kanMX4* | SB657 | 5A, 5D | This study |
| SY3045 | *MATa/α* [*PSI*^+^]^Sc4^ *ade1-14/ade1-14 his3Δ200/his3Δ200 trp1-289/ trp1-289 ura3-52/ura3-52::URA3::P_tet02_SUP35(G58D) leu2-3, 112/ leu2-3, 112 SUP35/sup35::kanMX4* | SB658 | 5A, 5D | This study |
