# Supplementary material for: A dominant-negative mutant inhibits multiple prion variants through a common mechanism
Source: PLoS Genet. 2017 Oct 30;13(10):e1007085. doi: 10.1371/journal.pgen.1007085 (PMC5679637; doi:10.1371/journal.pgen.1007085)
Supplement: S1 Text — (PDF) [file pgen.1007085.s009.pdf]

S1 Text: Mathematical Model

Introduction

We first describe the nucleated-polymerization model (NPM), introduced by Masel, Jensen and Nowak [4], which has formed the basis for modeling prion aggregate dynamics. The NPM - and various modifications - have been widely used to study prion phenotypes in yeast [1–3, 6]. We then discuss modifications of the NPM necessary to consider experiments in the presence of cycloheximide (CHX).

Nucleated Polymerization Model

The nucleated polymerization model (NPM) considers the temporal dynamics of soluble (monomeric) Sup35, $x(t)$, and aggregates of each discrete size, $y_i(t)$, for $i \geq n_0$ where n_0 is given and specifies the minimum stable aggregate size (nucleus). (These dynamics are graphically depicted in Figure 1.) Since aggregates

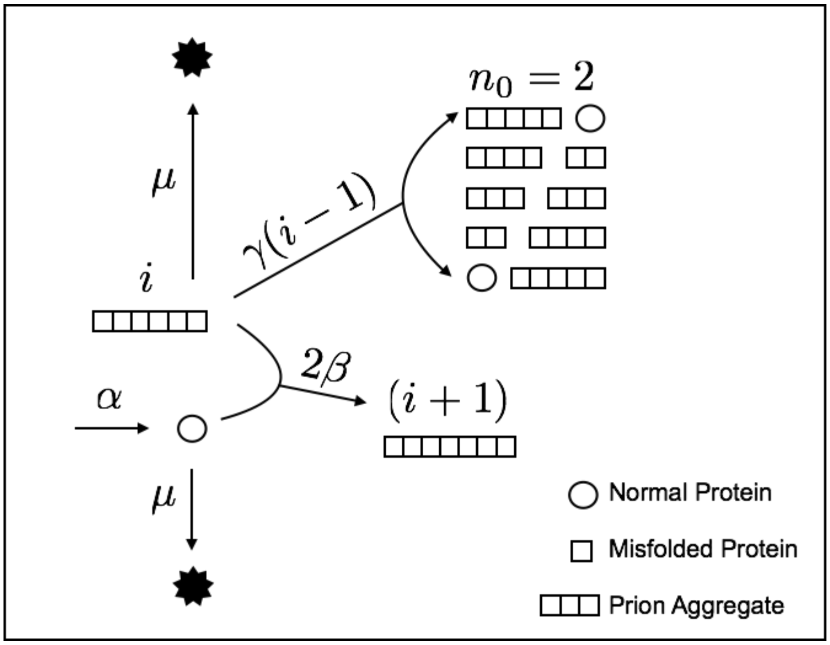

Figure 1: **Nucleated Polymerization Dynamics.** In the standard model of *in vivo* prion dynamics, normal protein (circles) interacts with prion aggregates (linear arrangements of squares). The kinetic rates for each reaction type - synthesis (α), conversion (β), fragmentation (γ), degradation/dilution (μ) - are shown for each biochemical reaction.

can be any finite size, the resulting system is an infinite set of ordinary differential equations (ODEs). However, there is a convenient moment-closure of the NPM which allows this infinite set of ODEs to be reduced to three time-varying values: $x(t)$ the concentration of soluble Sup35; $Y(t)$ the concentration of the number of aggregates and $Z(t)$ the concentration of aggregated protein [1, 4]. This formulation is referred to as the moment-closure because if $y_i(t)$ is the concentration of aggregates of size i then $Y(t)$ and $Z(t)$ correspond to the zeroth and first moments of the aggregate distribution:

$$Y(t) = \sum_{i=n_0}^{\infty} y_i(t) \text{ and } Z(t) = \sum_{i=n_0}^{\infty} i y_i(t). \tag{1}$$

The dynamics of aggregates in yeast depends on four biochemical processes and their respective rates: Sup35 synthesis (α) ; aggregate conversion (2β) ; fragmentation (γ); and dilution through cell-division (μ)

[5]. Under the standard assumption that conversion depends only on the ends of aggregates (and not their sizes) and fragmentation is linear in the number of monomer junctions for an aggregate, the moment-closure of the NPM has the following form:

$$\frac{dx}{dt} = \alpha - \mu x(t) - 2\beta x(t)Y(t) + \gamma n_0(n_0-1)Y(t) \tag{2}$$
$$\frac{dY}{dt} = \gamma Z(t) - (\mu + \gamma(2n_0-1))\,Y(t) \tag{3}$$
$$\frac{dZ}{dt} = 2\beta x(t)Y(t) - \mu Z(t) - \gamma n_0(n_0-1)Y(t). \tag{4}$$

As has been previously reported (see [1, 4] for example) the system has two steady-states. The first represents the disease-free steady-state (DF) where any prion aggregates would be cleared through the fragmentation (i.e., resolubilization) process or cell division/dilution:

$$x_{DF} = \alpha/\mu, \quad Z_{DF} = Y_{DF} = 0. \tag{5}$$

Alternatively, if prion aggregates are able to amplify fast enough to match loss from resolubilization or division/dilution the system reaches a stable configuration of aggregates (SS):

$$x_{SS} = \left(\frac{\gamma}{2\beta}\right)\left(n_0 + \frac{\mu}{\gamma}\right)\left(n_0 + \frac{\mu}{\gamma} - 1\right), \quad Z_{SS} = \frac{\alpha}{\mu} - x_{SS}, \quad Y_{SS} = Z_{SS}\left(2n_0 - 1 + \frac{\mu}{\gamma}\right)^{-1}. \tag{6}$$

The system is guaranteed to converge to the steady-state in Equation (6) whenever any initial aggregates are present and the basic reproductive number R_0 is larger than 1:

$$R_0 = \left(\frac{2\beta\gamma}{(\gamma n_0 + \mu)(\gamma(n_0-1)+\mu)}\right). \tag{7}$$

Otherwise the system will reach the disease-free steady state (Equation (5)).

Lastly, we point out two features of the NPM. First, for both steady-states the total amount of Sup35 in the system at steady-state is the same:

$$x_{SS} + Z_{SS} = \frac{\alpha}{\mu}, \quad x_{DF} + Z_{DF} = \frac{\alpha}{\mu}. \tag{8}$$

Second, when aggregates are preserved, the steady-state average aggregate size is given by:

$$\frac{Z_{ss}}{Y_{ss}} = 2n_0 - 1 + \frac{\mu}{\gamma}. \tag{9}$$

NPM in the Presence of Cycloheximide

When cells are treated with CHX two important changes occur that impact prion aggregate dynamics. First, the synthesis of new proteins is halted, and second cell division stops. When we incorporate these changes to the NPM (Equations (2)-(4)) to accurately reflect the system behavior under CHX treatment, we arrive at the following set of equations we call NPM+CHX:

$$\frac{dx}{dt} = -2\beta x(t)Y(t) + \gamma n_0(n_0-1)Y(t) \tag{10}$$
$$\frac{dY}{dt} = \gamma Z(t) - \gamma(2n_0-1)Y(t) \tag{11}$$
$$\frac{dZ}{dt} = 2\beta x(t)Y(t) - \gamma n_0(n_0-1)Y(t). \tag{12}$$

Notice that these equations look similar to the NPM except that the term for synthesis (α) was removed from the first equation, and the dilution term $(\mu x, \mu Y, \mu Z)$ was removed from each equation. In order to analyze

how treatment with CHX modifies the system, we assume the system has reached the steady-state of the NPM (i.e., cellular populations were growing for sufficiently long prior to CHX exposure). In this case, the total amount of Sup35 is at steady-state, and so for all time we have:

$$x(t) + Z(t) = \frac{\alpha}{\mu}.$$

As for the original NPM, this system has two steady-states, the disease free (which remains the same as above) and a new stable prion configuration C defined as follows:

$$x_C = \frac{\gamma n_0^2 - \gamma n_0}{2\beta}, \quad Z_C = \frac{\alpha}{\mu}, \quad Y_C = Z_C \left(2n_0 - 1\right)^{-1}. \tag{13}$$

By comparing the steady-states between the NPM and the NPM+CHX, we are able to observe several properties consistent with experimental observations (see main text). First, the ratio of steady-state soluble Sup35 in NPM+CHX is always smaller than for the original NPM. To see so, consider the following ratio:

$$\frac{x_C}{x_{SS}} = \frac{\left(\frac{\gamma n_0^2 - \gamma n_0}{2\beta}\right)}{\left(\frac{\gamma}{2\beta}\right)\left(n_0 + \frac{\mu}{\gamma}\right)\left(n_0 + \frac{\mu}{\gamma} - 1\right)} = \frac{(n_0 - 1)n_0}{(n_0 - 1)n_0 + \frac{\mu}{\gamma^2}\left(\mu + \gamma(2n_0 - 1)\right)}. \tag{14}$$

Because the numerator of this quantity is always smaller than the denominator, the resulting ratio will always be less than 1. The second term in the denominator controls how different these two quantities will be from one another (i.e., how far from 1 they will be). In addition, we note that this ratio depends only on three parameters (μ, γ, n_0). So, if we assume n_0 and μ to be the same between all prion strains studied, the strain with the **smallest** γ value will have the largest difference in this ratio, and the strain with the **largest** γ value will have the smallest difference in this ratio.

A similar comparison of Y_C to Y_{SS} shows that the number of aggregates under CHX will increase because aggregates will no longer be subject to dilution. In addition, we note that the average aggregate size is actually smaller under CHX

$$\frac{Z_{SS}}{Y_{SS}} = 2n_0 - 1 + \frac{\mu}{\gamma}, \quad \frac{Z_C}{Y_C} = \left(2n_0 - 1\right). \tag{15}$$

Finally, we note that while synthesis is inhibited, resolubilization of monomer through fragmentation, which occurs at rate $\gamma n_0(n_0 - 1)Y(t)$, will contribute to the soluble pool.

Parameter	Symbol	Value	Unit	Reference
Rate of Protein Synthesis	α	0.0154	μMmin^{-1}	[6]
Rate of Dilution from Cell Division	μ	0.0077	min^{-1}	[6]
Minimum Aggregate Size	n_0	5		[6]
Rate of Aggregate Conversion	β	0.158308	μM^{-1}min^{-1}	
Rate of Aggregate Fragmentation (Low)	γ	0.00221631	min^{-1}	
Rate of Aggregate Fragmentation (Medium)	γ	0.00443263	min^{-1}	
Rate of Aggregate Fragmentation (High)	γ	0.00886526	min^{-1}	

Table 1: Values for kinetic parameters used to calculate results presented in the main text. When possible, values were taken from prior studies. Otherwise, parameters were selected to fit the steady-state concentration of soluble protein and are intended to be representative.

References

[1] Jason K Davis and Suzanne S Sindi. A study in nucleated polymerization models of protein aggregation. *Applied Mathematics Letters*, 40:97–101, 2015.

[2] Jason K Davis and Suzanne S Sindi. A mathematical model of the dynamics of prion aggregates with chaperone-mediated fragmentation. *Journal of mathematical biology*, 72(6):1555–1578, 2016.

[3] Aaron Derdowski, Suzanne S Sindi, Courtney L Klaips, Susanne DiSalvo, and Tricia R Serio. A size threshold limits prion transmission and establishes phenotypic diversity. *Science*, 330(6004):680–683, 2010.

[4] Joanna Masel, Vincent A A Jansen, and Martin A. Nowak. Quantifying the kinetic parameters of prion replication. *Biophysical Chemistry*, 77(2-3):139–152, 1999.

[5] Suzanne S Sindi and Tricia R Serio. Prion dynamics and the quest for the genetic determinant in protein-only inheritance. *Current opinion in microbiology*, 12(6):623–630, 2009.

[6] Motomasa Tanaka, Sean R. Collins, Brandon H. Toyama, and Jonathan S. Weissman. The physical basis of how prion conformations determine strain phenotypes. *Nature*, 442(7102):585–9, 2006.
